# Nucleophagic Degradation of Progerin Ameliorates Defenestration in Liver Sinusoidal Endothelium Due to SIRT1-Mediated Deacetylation of Nuclear LC3

**DOI:** 10.3390/cells11233918

**Published:** 2022-12-03

**Authors:** Yangqiu Bai, Jinying Liu, Xiaoke Jiang, Xiuling Li, Bingyong Zhang, Xiaoying Luo

**Affiliations:** 1 Department of Gastroenterology, Henan Provincial People’s Hospital, Zhengzhou University People’s Hospital, Henan University People’s Hospital, No.7 Weiwu Road, Jinshui District, Zhengzhou 450003, China; 2 Microbiome Laboratory, Henan Provincial People’s Hospital, Zhengzhou University People’s Hospital, Henan University People’s Hospital, Zhengzhou 450003, China

**Keywords:** liver sinusoidal endothelial cell, defenestration, progerin, Sirtuin 1, LC3

## Abstract

Progerin, a permanently farnesylated prelamin A protein in cell nuclei, is potentially implicated in the defenestration of liver sinusoidal endothelial cells (LSECs) and liver fibrogenesis. Autophagy regulates the degradation of nuclear components, called nucleophagy, in response to damage. However, little is known about the role of nucleophagy in LSEC defenestration. Herein, we aim to dissect the underlying mechanism of progerin and nucleophagy in LSEC phenotype. We found an abnormal accumulation of progerin and a loss of SIRT1 in the nucleus of intrahepatic cells in human fibrotic liver tissue. In vivo, nuclear progerin abnormally accumulated in defenestrated LSECs, along with a depletion of SIRT1 and Cav-1 during liver fibrogenesis, whereas these effects were reversed by the overexpression of SIRT1 with the adenovirus vector. In vitro, H_2_O_2_ induced the excessive accumulation of progeirn, with the depletion of Lamin B1 and Cav-1 to aggravate LSEC defenestration. NAC and mito-TEMPO, classical antioxidants, inhibited NOX2- and NOX4-dependent oxidative stress to improve the depletion of Lamin B1 and Cav-1 and promoted progerin-related nucleophagy, leading to a reverse in H_2_O_2_-induced LSEC defenestration. However, rapamycin aggravated the H_2_O_2_-induced depletion of Lamin B1 and Cav-1 due to excessive autophagy, despite promoting progerin nucleophagic degradation. In addition, overexpressing SIRT1 with the adenovirus vector inhibited oxidative stress to rescue the production of Lamin B1 and Cav-1. Moreover, the SIRT1-mediated deacetylation of nuclear LC3 promoted progerin nucleophagic degradation and subsequently inhibited the degradation of Lamin B1 and Cav-1, as well as improved F-actin remodeling, contributing to maintaining LSEC fenestrae. Hence, our findings indicate a new strategy for reversing LSEC defenestration by promoting progerin clearance via the SIRT1-mediated deacetylation of nuclear LC3.

## 1. Introduction

Liver sinusoidal endothelial cells (LSECs) are highly specialized endothelial cells, which contain open fenestrae without a diaphragm normally. The defenestration and capillarization of LSECs activate hepatic stellate cells (HSCs) and promote liver fibrosis, whereas maintaining LSEC fenestrae and reversing their capillarization can prevent the progression of liver fibrosis [1]. However, the mechanisms underlying LSEC defenestration and how to recover LSEC phenotype and function remain elusive.

Previous studies by us and others have confirmed that cellular senescence is involved in liver fibrosis [2,3,4]. Furthermore, we indicated that stress-induced premature senescence could accelerate LSEC defenestration and liver fibrosis due to the abnormal accumulation of progerin [2,5]. Progerin, a special marker of premature senescence, is a permanently farnesylated prelamin A protein in cell nuclei. Progerin can induce the alteration of nuclear morphology and promote cells into an early onset of aging phenotype [6,7]. An inference may be made on progerin clearance being a potential treatment for protecting LSEC phenotype and function, which is a new area of investigation.

Autophagy, an intracellular recycling system, self-digests damaged organelles and proteins to maintain cellular homeostasis. There is increasing evidence demonstrating that autophagy plays a key role in LSEC morphology and liver diseases [8,9]. Most studies largely focus on the autophagy of cytoplasmic proteins, but little is known about the effects of autophagy of nuclear components (called nucleophagy) on LSEC phenotype and liver fibrosis.

Sirtuin 1 (SIRT1), a kind of nicotinamide adenine dinucleotide (NAD^+^)-dependent histone deacetylase, confers protection against liver aging and liver diseases [10,11]. SIRT1 has recently been reported as a new selective protein, linked to nucleophagy and senescence intervention [12]. This led us to test the hypothesis that SIRT1-regulated progerin degradation, which was potentially mediated by nucleophagy, could protect against LSEC defenestration and liver fibrosis.

## 2. Materials and Methods

### 2.1. Antibodies and Reagents

The primary antibodies included anti-vWF (Santa Cruz, #sc-365712), anti-COL 1A1 (Proteintech, #14695-1-AP), anti-progerin (Santa Cruz, #sc-81611), anti-Lamin A/C (Cell Signaling Technology, #4777S), anti-Lamin B1 (Proteintech, #66095-1-Ig), anti-SIRT1 (Abcam, #ab110304), anti-LC3B (Abcam, #ab48394), anti-acetyl Lysine (Abcam, #ab22550), anti-Caveolin-1 (Cav-1) (Abcam, #ab32577), anti-Histone H3 (Proteintech, #17168-1-AP), and anti-GAPDH (Proteintech, #60004-1). The secondary antibodies included Cy3-labeled goat anti-mouse IgG (H+L) (Beyotime, #a0521), FITC-labeled goat anti-rabbit IgG (H+L) (Beyotime, #a0562), HRP-conjugated Affinipure Goat Anti-Mouse IgG(H+L) (Proteintech, #SA00001-1), and HRP-conjugated Affinipure Goat Anti-Rabbit IgG(H+L) (Proteintech, #SA00001-2). 

The reagents used included carbon tetrachloride (CCl_4_) (Sigma-Aldrich, #56-23-5), hydrogen peroxide (H_2_O_2_) (Sigma-Aldrich, #1.07298), N-Acetylcysteine (NAC) (MedChemExpress, #616-91-1), mitochondria 2,2,6,6-tetramethylpiperidinooxy (mito-TEMPO) (MedChemExpress, #1334850-99-5), 3-Methyladenine (3-MA) (Sigma-Aldrich, #S2767), rapamycin (sirolimus) (Sigma-Aldrich, #S1039), MCDB131 (Gibco, #10372019), 1640 (Gibco, #11875101), and fetal bovine serum (FBS) (Biological Industries, #04-007-1A).

### 2.2. Clinical Samples

The clinical research was approved by the Committee on the Ethics of Henan Provincial People’s Hospital (Zhengzhou, China), and the ethical approval code was 2021-30. Patients were recruited from our hospital and signed the informed written consent. Plasma samples were obtained from 38 patients with liver fibrosis and 38 age-matched controls. Fibrotic liver biopsy specimens were obtained from 9 of the above patients with liver fibrosis; and adjacent normal liver specimens were obtained from 8 of the above patients in the control group, who underwent a partial liver resection due to hepatic hemangioma.

### 2.3. Experimental Rat Models and Treatments

Male Sprague–Dawley (SD) rats (6 weeks old, 180–200 g) were provided by the Beijing Vital River Laboratory Animal Technology Co., Ltd. in China. The quality certificate number of the laboratory animals was 11400700349237. The rat experiments were approved by the Committee on the Ethics of Animal Experiments of Zhengzhou University (Zhengzhou, China), and the ethical approval code was 20210515. Rats were housed under the 12 h:12 h light/dark cycle at 22–24 °C. 

To establish the early stage of CCl_4_-induced liver fibrosis rat models, rats were subjected to intraperitoneal injection of 40% CCl_4_-olive oil solution at 2 mL/kg body weight, twice a week for 28 days [13]. Rats were randomly sacrificed on day 0, day 3, day 6, day 14, and day 28 (N = 6 per group). 

Next, to further investigate the effects of SIRT1 on LSEC fenestrae and liver fibrosis, the rat models were administrated with GFP-tagged SIRT1 adenovirus vectors to overexpress SIRT1. Rats were randomly divided into four groups (N = 12 per group): the vehicle group, the CCl_4_ group, the CCl_4_+AV-CTR group, and the CCl_4_+AV-SIRT1 group. Rats of the CCl_4_+AV-SIRT1 group were pre-injected in the caudal vein with the GFP-tagged SIRT1 adenovirus vectors (1 × 10^11^ per rat), whereas rats of the CCl_4_+AV-CTR group were pre-injected in the caudal vein with the GFP-tagged blank vectors. The transfection efficiency, detected by immunofluorescent (IF) staining, was about 70%. Subsequently, rats of the CCl_4_ group, the CCl_4_+AV-CTR group, and the CCl_4_+AV-SIRT1 group were injected intraperitoneally with 40% CCl_4_-olive oil solution to establish CCl_4_-induced liver fibrosis models as indicated above. Meanwhile, rats of the vehicle group were given the same volume of olive oil. On day 6 and day 28, six rats in each group were randomly sacrificed. No rats died accidentally in the experiment. The adenovirus vectors were provided by Hanbio AdenoVector Institute (Shanghai, China). The SIRT1 sequences used were: sense (5′-CGGGCCCTCTAGACTCGAGCGGCCGCATGATTGGCACCGATCCTC-3′).

### 2.4. Histological Analysis and Immunohistochemistry (IHC)

Fresh fibrotic liver biopsy specimens of patients and liver tissue of rat models were fixed in 4% paraformaldehyde and then embedded in paraffin blocks. The embedded tissue was cut with the paraffin microtome (Leica, #RM2235, Germany).

Hepatic paraffin sections (4 μm) of patients and rat models were prepared for hematoxylin-eosin (HE) staining and Masson staining. The sections were visualized with a microscope (BX51, Olympus, Japan). The ISHAK score was used for the assessment of liver histological inflammation and fibrosis stage.

In addition, hepatic paraffin sections (3 μm) of patients and rats were prepared for the immunohistochemical detection of progerin, SIRT1, vWF, and COL1A1. The primary antibodies included anti-progerin (1:50), anti-SIRT1 (1:200), anti-vWF (1:200), and anti-COL1A1 (1:200). Subsequently, the sections were exposed to the HRP-antibody colored with DAB. The IHC staining was visualized by the microscope (BX51, Olympus, Japan). The area densities of the IHC staining of progerin, SIRT1, vWF, and COL1A1 were semi-quantified with Image J V1.8.0 software.

### 2.5. Rat Primary LSECs Isolation, Culture, and Treatments

Rat primary LSECs of the rat models were isolated by differential centrifugation with intrahepatic cells’ density gradient and sorted by the flow cytometry sorter (BD, FACSAria III), according to the methods of our previous research [13].

In addition, in vitro, normal rat primary LSECs were isolated from normal male SD rats (6 weeks old, 180–200 g), according to the methods indicated above. Primary LSECs and their fenestrae were identified by scanning electron microscope (SEM). Primary LSECs were cultured with the medium, which comprised 20% FBS, 40% 1640, and 40% MCDB131. Primary LSECs were stimulated by H_2_O_2_ (10 μM) with a time gradient (24 h and 48 h) and administrated with NAC (1 mM), mito-TEMPO (100 U/mL), 3-MA (10 μM), and rapamycin (10 nM). The experiment was repeated three times.

### 2.6. Small Interfering RNA (siRNA) and Adenoviral Vector Transfections

Normal rat primary LSECs were pre-transfected with LC3B siRNA and SIRT1 siRNA to knockdown LC3B and SIRT1 and then stimulated by H_2_O_2_ (10 μM). The transfection efficiency was about 75%. LC3B siRNA and SIRT1 siRNA were produced by the Ribobio Company (Guangzhou, China). The following LC3B siRNA sequences were used: sense (5′-GCGAACTCATCAAGATAAT-3′). The following SIRT1 siRNA sequences were used: sense (5′-CCTCAAGCCATGTTCGATA-3′). The experiment was repeated three times.

To overexpress SIRT1, normal rat primary LSECs were transfected with the recombinant Flag-tagged SIRT1 adenovirus vector, which was produced by the Hanbio AdenoVector Institute (Shanghai, China). The transfection efficiency was about 70–75%. The SIRT1 sequences were used: sense (5′-CGGGCCCTCTAGACTCGAGCGGCCGCATGATTGGCACCGATCCTC-3′). The experiment was repeated three times.

### 2.7. Scanning Electron Microscopy (SEM)

Hepatic sinusoidal endothelium in the liver tissue (size: 1 mm^3^) of rat models and rat primary LSECs in cell slides were fixed with 2.5% glutaldehyde, dehydrated, and coated with gold. LSEC fenestrae were observed with SEM at a 15-kV acceleration voltage. The experiment was repeated more than three times.

### 2.8. RT-qPCR

Total RNA of the peripheral blood granulocytes of patients and rat primary LSECs (10^6^) was extracted with Trizol and reverse-transcribed with PrimeScriptTM RT Maseter Mix (Takara, #RR036A). Real-time PCR was performed with SYBR Premix Ex TaqTM IIz (Takara, #RR820A), using ROCHE LightCycler^®^480. The samples were analyzed using the 2^-∆∆Ct^ method from the Ct values of the respective RNAs (progerin, NOX2, NOX4, *LMNA*, Lamin B1, and Cav-1) relative to the housekeeping gene GAPDH. The primer sequences are displayed in the Supplementary Materials. The experiment was repeated more than three times.

### 2.9. Renilla Luciferase Reporter Gene Assay of LMNA

The *LMNA* (Rattus norvegicus (Norway rat), Gene ID: 60374) promoter region (P1 covers 1500 bp upstream of the transcription start site; P2 is from 3000 bp to 1500 bp upstream of the transcription start site) was cloned in to pmirGLO (Promega). Primary LSECs were co-transfected with 1 ug LMNA promoter luciferase plasmid and 100 ng Renilla luciferase reporter control plasmid (Promega). After transfection for 48 h, the luciferase activity of the above treated-LSECs and its renilla luciferase activity were measured with the Dual-Luciferase Reporter Assay System and normalized by renilla luciferase activity.

### 2.10. Immunofluorenscent (IF) Staining and Immunocytochemistry (ICC)

Hepatic paraffin sections (3 μm) of CCl_4_-induced liver fibrosis rat models were prepared for the IF staining for progerin, vWF, and LC3B. The primary antibodies included anti-progerin (1:50), anti-vWF (1:200), and anti-LC3B (1:200). Subsequently, the sections were exposed to the secondary antibodies and DAPI. The sections were visualized with the fluorescence microscope.

The treated LSECs in confocal dishes were fixed with 4% paraformaldehyde and then permeabilized with 0.1% Triton X-100 and blocked with 3% BSA. Next, LSECs were incubated with primary antibodies, including anti-acetyl Lysine (1:200), anti-progerin (1:50), and anti-LC3B (1:200), followed by the secondary antibodies and DAPI. Lastly, LSECs were stained with a phallotoxin (Thermo, #A22287 and #T7471) to detect F-actin. The positive cells were observed by the confocal microscopy and quantified by Image J V1.8.0 software. The experiment was repeated three times.

### 2.11. Hydrogen Peroxide Assay

Primary LSECs were isolated from the above rat models. The H_2_O_2_ content of LSECs was measured by the Hydrogen Peroxide Assay Kit (Beyotime, #S0038), according to the manufacture’s specifications. The OD value was detected by absorption spectroscopy (562 nm). The experiment was repeated three times.

### 2.12. Intracellular ROS and Mitochondrial ROS (mito-ROS)

According to the manufacture’s protocol, primary LSECs of rat models and the treated LSECs in vitro were incubated with fluorescence probes DCFH DA (Beyotime, #S0033S) and a MitoSOX^TM^ Red Mitochondrial Superoxide Indicator (Thermo, #M36008) and detected by flow cytometer (BD, FACSCanto II) to analyze ROS and mito-ROS. The experiment was repeated three times.

### 2.13. Extraction of Nuclear and Cytoplasmic Protein of Rat Primary LSECs

The treated primary LSECs (10^7^ cells/group) were lysed by lysis solution and then nuclear and cytoplasmic protein was extracted with the Nuclear and Cytoplasmic Protein Extraction Kit (Beyotime, #P0028), according to the manufacture’s protocol.

### 2.14. Western Blotting

Liver tissue of patients and rats and rat primary LSECs were lysed in the lysis buffer, which contained the protease cocktail inhibitor and PMSF. The protein was centrifuged at 12,000× *g* at 4 °C for 15 min, and its concentration was detected by the BCA protein assay kit (Beyotime, #P0012S). The protein expression was detected by western blotting. Additionally, the nuclear and cytoplasmic proteins of primary LSECs were also detected by western blotting after the detection of their concentration. The primary antibodies included anti-α-SMA (1:1000), anti-vWF (1:500), anti-SIRT1 (1:1000), anti-progerin (1:50), anti-Lamin A/C (1:1000), anti-Lamin B1 (1:1000), anti-Cav-1 (1:1000), anti-NOX2 (1:1000), anti-NOX4 (1:1000), anti-LC3B (1:1000), anti-GAPDH (1:1000), and anti-Histone H3 (1:1000). The secondary antibodies were HRP-conjugated Affinipure Goat Anti-Mouse IgG (H+L) (1:10,000) and HRP-conjugated Affinipure Goat Anti-Rabbit IgG (H+L) (1:10,000). The bands were observed by the Pierce™ ECL Western Blotting Substrate. The experiment was repeated more than three times.

### 2.15. Co-Immunoprecipitation (Co-IP)

The interaction of nuclear LC3B with acetyl Lysine, progerin, Lamin B1, and Cav-1, as well as the interaction of cytoplasmic LC3B with Cav-1 of the treated primary LSECs were detected by the co-IP assay. IP and immunoblotting (IB) were performed as previously described [13]. The antibodies for IP included anti-LC3B and non-specific IgG, and the antibodies for IB included anti-acetyl Lysine, anti-progerin, anti-Lamin A/C, anti-Lamin B1, and anti-Cav-1. The experiment was repeated more than three times.

### 2.16. Statistics

All data were analyzed by SPSS 20.0 software and reported as the mean ± SD. In the statistical analysis of two groups, a two-tailed Student’s *t*-test was performed. In statistical analysis of more than two groups, one-way ANOVA was utilized. A *p* < 0.05 was considered significant.

## 3. Results

### 3.1. Progerin Is Elevated in Human Liver Fibrosis, along with Depletion of SIRT1

**Figure 1:**
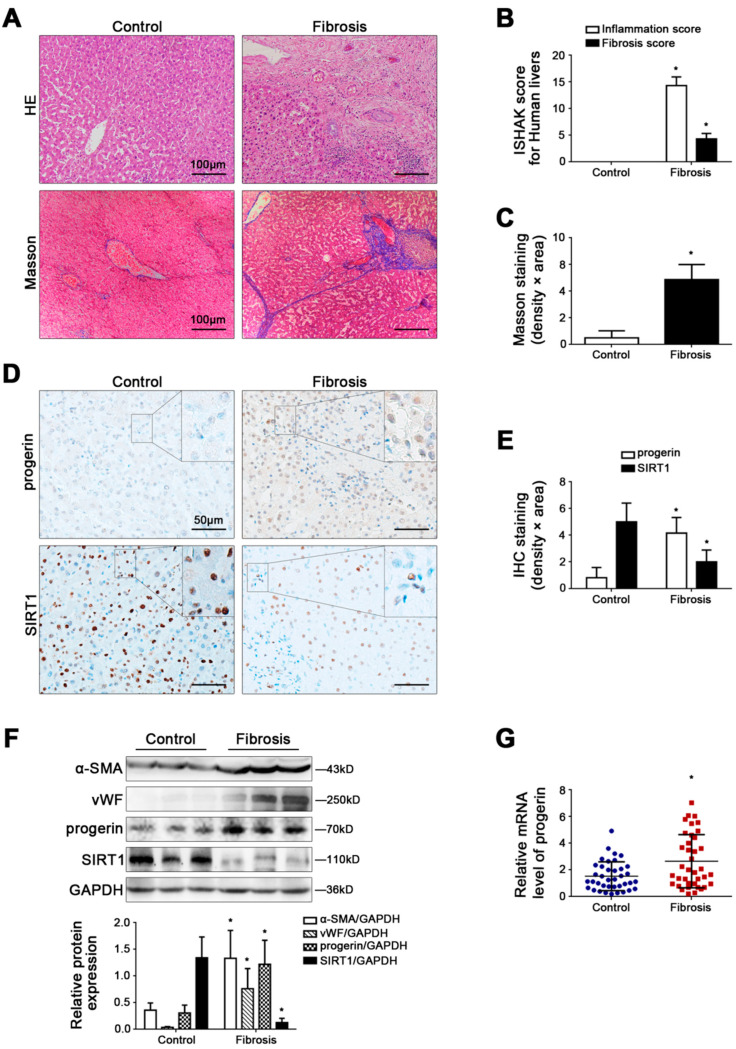
Progerin is elevated in human liver fibrosis, along with depletion of SIRT1. (**A**) HE and Masson staining in human liver specimens (Scale bar: 100 μm). (**B**) The quantified analysis of liver inflammation and fibrosis with ISHAK score in the graph. * *p* < 0.05 versus the control group. (**C**) The semi-quantified analysis for the area density of Masson staining in the graph. * *p* < 0.05 versus the control group. (**D**) IHC staining for progerin and SIRT1 in human liver specimens (scale bar: 50 μm). (**E**) The semi-quantified analysis for the area density of IHC staining of progerin and SIRT1 in the graph. * *p* < 0.05 versus the control group. (**F**) Representative immunoblots of α-SMA, vWF, progerin, and SIRT1 in human liver tissue. The relative protein levels were quantified in the graph (below). * *p* < 0.05 versus the control group. (**G**) RT-qPCR analysis for progerin mRNA level in human peripheral blood granulocytes. * *p* < 0.05 versus the control group.

In human liver fibrotic tissue, the inflammation and fibrosis score, the area density of Masson staining, as well as the protein expression of α-SMA and vWF increased (Figure 1A–C,F). Intriguingly, the IHC staining and western blotting displayed that progerin was highly expressed in the nucleus of intrahepatic cells in human liver fibrotic tissue (Figure 1D–F). Moreover, compared to the control group, the mRNA level of progerin in the peripheral blood granulocytes of patients was drastically enhanced in the liver fibrosis group (*p* = 0.003) (Figure 1G). However, the IHC staining and western blotting showed low expression of SIRT1 in the nucleus of intrahepatic cells in human liver fibrotic tissue (Figure 1D–F). The findings suggest that augment of progerin and the loss of SIRT1 are implicated in liver fibrogenesis.

### 3.2. Abnormal Accumulation of Progerin and Cav-1-Related Autophagy Emerge in Defenestrated and Capillarized Hepatic Sinusoidal Endothelium, along with Loss of SIRT1

**Figure 2:**
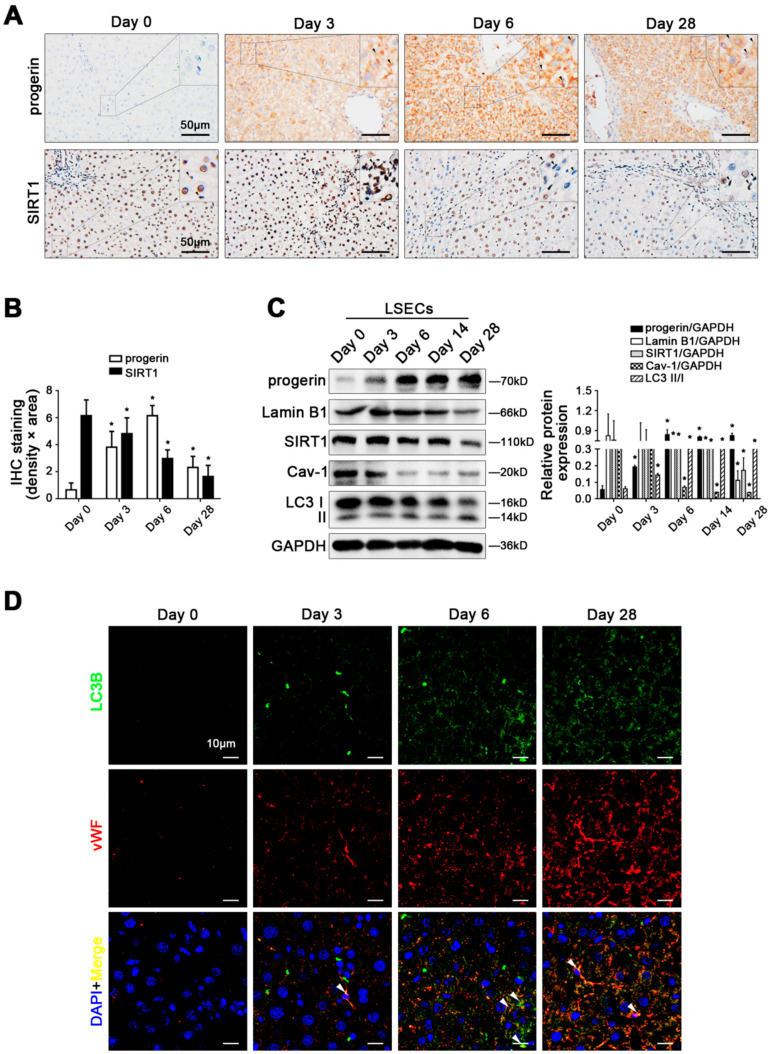
Abnormal accumulation of progerin and Cav-1-related autophagy emerge in defenestrated and capillarized hepatic sinusoidal endothelium, along with loss of SIRT1. (**A**) IHC staining of progerin and SIRT1 in liver tissue of CCl_4_-induced rat models at different time points (day 0, day 3, day 6, and day 28) (scale bar: 50 μm). The black triangles indicate progerin- and SIRT1-positive LSECs. (**B**) The semi-quantified analysis for the area density of IHC staining of progerin and SIRT1 in the graph. * *p* < 0.05 versus the day 0 group. (**C**) Representative immunoblots of progerin, Lamin B1, SIRT1, Cav-1, and LC3 II/I in primary LSECs, which were isolated from CCl_4_-induced rat models at different time points (day 0, day 3, day 6, day 14, and day 28). The relative protein expression was quantified in the graph (right). * *p* < 0.05 versus the day 0 group. (**D**) The co-localization of LC3B (green) with vWF (red) in liver tissue of CCl_4_-induced rat models at different time points (day 0, day 3, day 6, and day 28) (scale bar: 10 μm). Nuclei are shown by DAPI (blue). N = 6 per group.

We previously revealed the defenestration in LSECs on the 6th day in CCl_4_-induced rat liver fibrosis models [13]. As indicated in Supplementary Figure S1A–D, HE staining, Masson staining, and the IHC staining showed an increase in COL1A1 and vWF protein expression in the first stage of CCl_4_-induced liver fibrosis. Interestingly, during CCl_4_-induced liver fibrogenesis, there was a time-dependent elevation of progerin protein expression in primary LSECs, along with an increase in ROS and mito-ROS (Figure 2A–C and Figure S1E). In contrast, the protein levels of Lamin B1 and SIRT1 were decreased in the primary LSECs of CCl_4_-induced rat models (Figure 2C). Our previous study demonstrated the involvement of Cav-1 autophagic degradation in LSECs defenestration during liver fibrogenesis [13,14]. Indeed, there was a time-dependent decline in Cav-1 protein expression in the primary LSECs, along with an augmentation of LC3 II/I expression during CCl_4_-induced liver fibrogenesis (Figure 2C). Moreover, the immunofluorescence displayed that LC3 highly expressed in vWF-positive liver sinusoidal endothelium in CCl_4_-induced rat models (Figure 2D). Hence, this data indicated that progerin abnormally accumulated in defenestrated and capillarized hepatic sinusoidal endothelium, along with the depletion of SIRT1 and Cav-1 in liver fibrogenesis.

### 3.3. SIRT1 Gene Transfer to CCl_4_-Induced Rat Models Alleviates LSECs Defenestration and Liver Fibrosis by Reducing Progerin and Reversing Cav-1 Degradation

**Figure 3:**
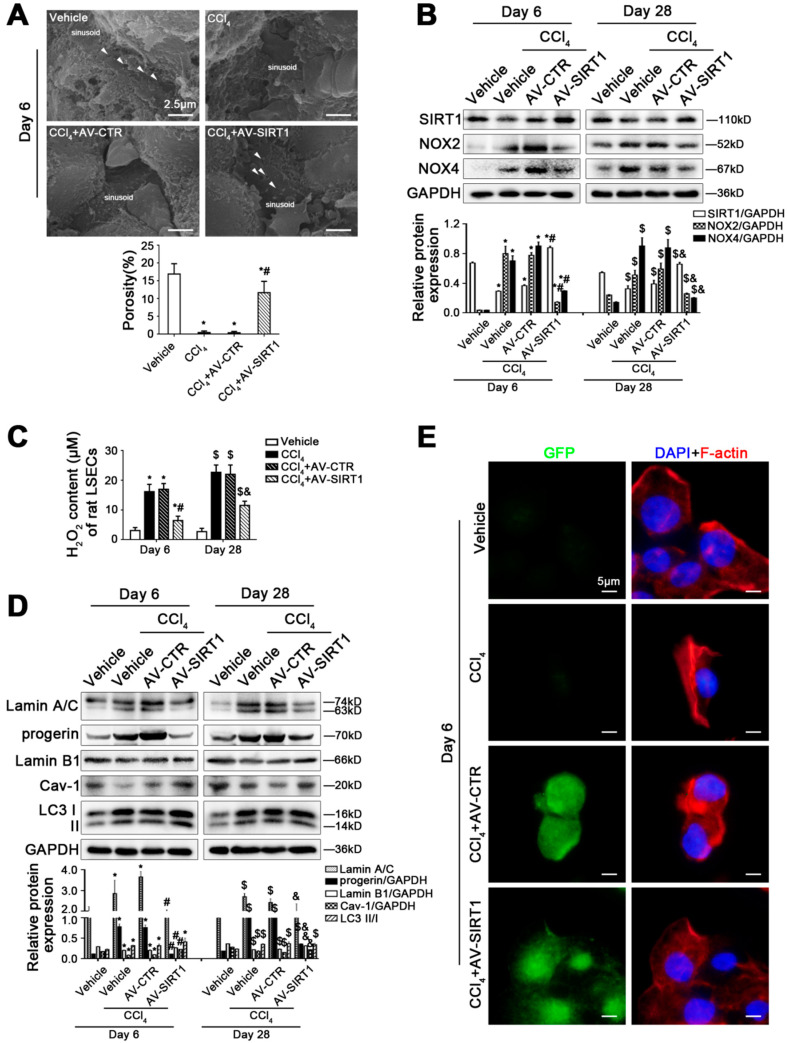
SIRT1 gene transfer to CCl_4_-induced rat models alleviates LSEC defenestration and liver fibrosis through reducing progerin and reversing Cav-1 degradation. (**A**) The magnification of SEM for fenestrae in the liver sinusoidal endothelium of CCl_4_-induced rat models in the four groups (vehicle, CCl_4_, CCl_4_+AV-CTR, CCl_4_+AV-SIRT1) on day 6 (Scale bar: 2.5 μm). The white triangles indicate fenestrae in liver sinusoidal endothelium. The porosity is quantified in the graph (below). * *p* < 0.05 versus the vehicle group; ^#^ *p* < 0.05 versus the CCl_4_+AV-CTR group. (**B**) Representative immunoblots of SIRT1, NOX2, and NOX4 in primary LSECs, isolated from CCl_4_-induced rat models on day 6 and day 28. The relative protein expression was quantified in the graph (below). * *p* < 0.05 versus the vehicle group on day 6; ^#^ *p* < 0.05 versus the CCl_4_+AV-CTR group on day 6; ^$^ *p* < 0.05 versus the vehicle group on day 28; ^&^ *p* < 0.05 versus the CCl_4_+AV-CTR group on day 28. (**C**) The H_2_O_2_ content of primary LSECs, isolated from CCl_4_-induced rat models on day 6 and day 28. * *p* < 0.05 versus the vehicle group on day 6; ^#^ *p* < 0.05 versus the CCl_4_+AV-CTR group on day 6; ^$^ *p* < 0.05 versus the vehicle group on day 28; ^&^ *p* < 0.05 versus the CCl_4_+AV-CTR group on day 28. (**D**) Representative immunoblots of Lamin A/C, progerin, Lamin B1, Cav-1, and LC3 II/I in primary LSECs, isolated from CCl_4_-induced rat models on day 6 and day 28. The relative protein expression is quantified in the graph (below). * *p* < 0.05 versus the vehicle group on day 6; ^#^ *p* < 0.05 versus the CCl_4_+AV-CTR group on day 6; ^$^ *p* < 0.05 versus the vehicle group on day 28; ^&^ *p* < 0.05 versus the CCl_4_+AV-CTR group on day 28. (**E**) ICC staining of F-actin (red) in primary LSECs, isolated from CCl_4_-induced rat models on day 6 (Scale bar: 5 μm). Adenovirus vectors are shown by GFP (green). Nuclei are shown by DAPI (blue). N = 12 per group.

To investigate the interplay between SIRT1, progerin, and Cav-1-related autophagy in LSEC defenestration during liver fibrogenesis, we employed CCl_4_-induced rat models, which were administrated with GFP-tagged SIRT1 adenovirus vector (called AV-SIRT1) or the blank vector (called AV-CTR) in advance. The transfection efficiency was about 70%, which was detected by immunofluorescence. As shown in Figure 3A and Figure S2A, the data of SEM and HE staining showed that the overexpression of SIRT1 with the adenovirus vector reversed CCl_4_-induced LSEC defenestration on the 6th day and alleviated the first stage of liver fibrosis on the 28th day. Compared to the vehicle group on the 6th and 28th day, the protein levels of NOX2 and NOX4 and the H_2_O_2_ content were elevated in primary LSECs in the CCl_4_ group and the CCl_4_+AV-CTR group, whereas these effects were reduced in the CCl_4_+AV-SIRT1 group (Figure 3B,C), suggesting that overexpressing SIRT1 could inhibit NOX2- or NOX4-dependent oxidative stress. In addition, compared to the vehicle group on the 6th and 28th day, the protein expression of Lamin A/C and progerin increased in primary LSECs of the CCl_4_ group and the CCl_4_+AV-CTR group, along with a decrease in Lamin B1. However, the overexpression of SIRT1 with the adenovirus vector lessened the protein levels of Lamin A/C and progerin but recovered Lamin B1 protein expression (Figure 3D and Figure S2B). Unexpectedly, although LC3 II/I protein expression was elevated in the primary LSECs in the CCl_4_ group, the CCl_4_+AV-CTR group, and the CCl_4_+AV-SIRT1 group, the CCl_4_-induced loss of Cav-1 in the primary LSECs was rescued by the overexpression of SIRT1 with the adenovirus vector (Figure 3D and Figure S2C). The data implied that overexpressing SIRT1 might increase Cav-1 generation. Further, the activation of autophagy, induced by overexpressing SIRT1, might be implicated in progerin clearance rather than Cav-1 degradation. In addition, immunofluorescence showed that compared to the vehicle group, F-actin aberrantly accumulated in the nucleus and perinuclear area in primary LSECs in the CCl_4_ group and the AV-CTR+CCl_4_ group, whereas F-actin distributed uniform cytoplasm in the AV-SIRT1+CCl_4_ group (Figure 3E). These results implied that the overexpression of SIRT1 with the adenovirus vector might promote progerin clearance, inhibit the loss of Lamin B1 and Cav-1, and improve F-actin remodeling to attenuate LSEC defenestration and liver fibrosis.

### 3.4. H_2_O_2_ Induces Excessive Accumulation of Progeirn, with Depletion of Lamin B1 and Cav-1 to Aggravate LSEC Defenestration

In vitro, rat primary LSECs were cultured without growth factors for five days. As shown in Supplementary Figure S3A,B, in the progression of LSEC defenestration, there was a time-dependent increase in progerin in the nucleus of the primary LSECs, along with an augmentation of LC3 II/I in the nucleus and cytoplasm; in contrast, nuclear Lamin B1 expression and nuclear and cytoplasmic Cav-1 expression were down-regulated from day 1 to day 5. Furthermore, the co-IP assay showed that during LSEC defenestration, elevated acetyl Lysine, progerin, Lamin B1, and Cav-1 co-precipitated with LC3B in the nucleus in primary LSECs from day 1 to day 5 (Supplementary Figure S3C). In addition, more Cav-1 co-precipitated with LC3B in the cytoplasm of the primary LSECs from day 1 to day 5 (Supplementary Figure S3D). The data suggested that excessive accumulation of progerin, along with Lamin B1-related nucleophagy and Cav-1 autophagic degradation, was potentially implicated in LSEC defenestration.

**Figure 4:**
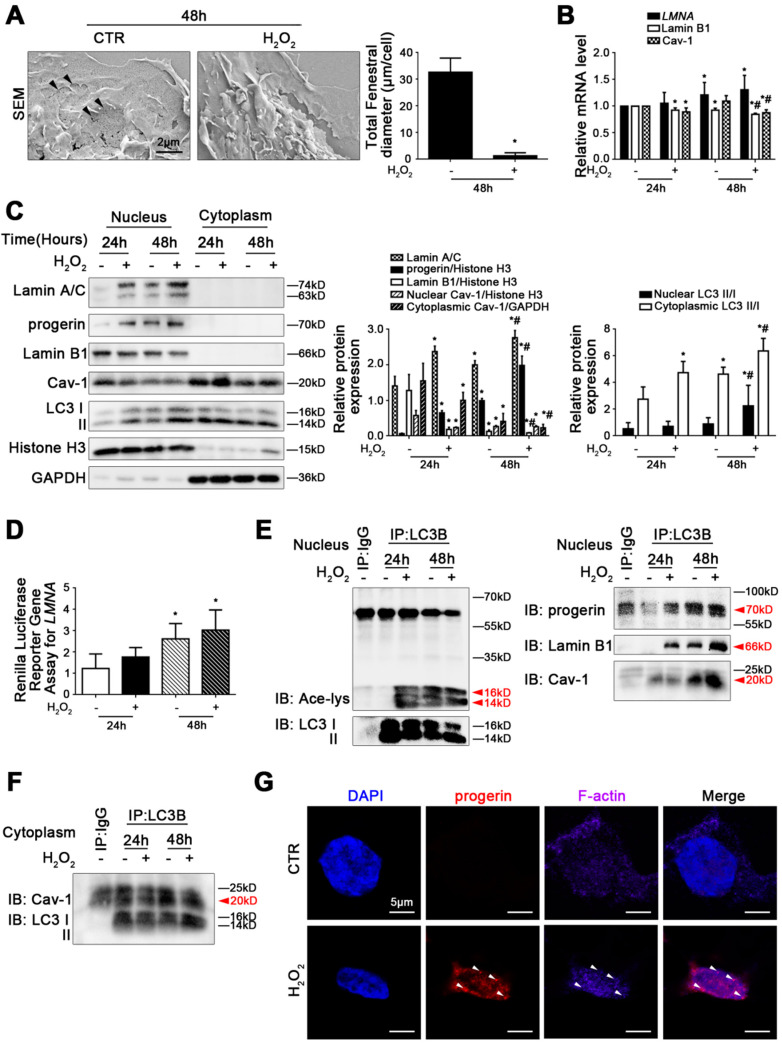
H_2_O_2_ induces excessive accumulation of progeirn, with depletion of Lamin B1 and Cav-1 to aggravate LSEC defenestration. Fresh rat primary LSECs, isolated from normal male SD rats, were treated with H_2_O_2_ (10 μM) in vitro for 48 h. (**A**) The magnification of SEM for fenestrae in primary LSECs in 48 h (scale bar: 2 μm). The black triangles indicate fenestrae in LSECs. The total fenestral diameter is quantified in the graph (right). * *p* < 0.05 versus the control group in 48 h. (**B**) RT-qPCR analysis for mRNA levels of *LMNA*, Lamin B1, and Cav-1 of primary LSECs in 24 h and 48 h. * *p* < 0.05 versus the control group in 24 h; ^#^ *p* < 0.05 versus the control group in 48 h. (**C**) Representative immunoblots of Lamin A/C, progerin, Lamin B1, Cav-1, and LC3 II/I in nucleus and cytoplasm of primary LSECs in 24 h and 48 h. The relative protein expression and LC3 II/I expression in nucleus and cytoplasm are quantified in the two graphs (right). * *p* < 0.05 versus the control group in 24 h; ^#^ *p* < 0.05 versus the control group in 48 h. (**D**) Renilla Luciferase Reporter Gene Assay for *LMNA* promoter activity of primary LSECs in 24 h and 48 h. * *p* < 0.05 versus the control group in 24 h. (**E**) The interaction of nuclear LC3B with acetyl Lysine, progerin, Lamin B1, and Cav-1 of primary LSECs in 24 h and 48 h was detected by the co-IP assay. Nuclear LC3B in primary LSECs was individually immunoprecipitated, and subsequently, acetyl Lysine, progerin, Lamin B1, Cav-1, and LC3 II/I in nucleus of primary LSECs were subjected to immunoblotting analysis. (**F**) The interaction of cytoplasmic LC3B with Cav-1 of primary LSECs in 24 h and 48 h was detected by the co-IP assay. Cytoplasmic LC3B in primary LSECs was individually immunoprecipitated, and subsequently, Cav-1 and LC3 II/I in cytoplasm of primary LSECs were subjected to immunoblotting analysis. (**G**) The immunocytochemical co-localization of progerin (red) with F-actin (purple) of primary LSECs in 48 h (Scale bar: 5 μm). Nuclei are shown by DAPI (blue).

Further, we treated rat primary LSECs with H_2_O_2_ for 48 h In vitro. As we expected, the magnification of SEM, the mRNA levels, and the protein expression of NOX2 and NOX4, as well as the ROS and mito-ROS levels, showed that H_2_O_2_-induced oxidative stress activated the NOX2- and NOX4-dependent signaling pathway to bring about LSEC defenestration (Figure 4A and Figure S4A–C). Interestingly, the RT-qPCR experiment displayed that there was a time-dependent increase in the *LMNA* mRNA level from 24 h to 48 h (Figure 4B). Meanwhile, western blotting showed that the protein levels of progerin and Lamin A/C in the nucleus, which could be transcribed from the *LMNA* gene, were also up-regulated from 24 h to 48 h (Figure 4C). The data indicated that the fenestrae of the primary LSECs, cultured in vitro, emerged in contraction or even disappeared, along with the augmentation of progerin and Lamin A/C. However, although there was no difference in *LMNA* mRNA levels between the control group and the H_2_O_2_ group in 24 h and 48 h, compared to the concurrent control group, the protein expression of Lamin A/C and progerin in the nucleus of the H_2_O_2_-treated LSECs was still elevated (Figure 4B,C). Furthermore, the renilla luciferase reporter gene assay displayed that there was no difference in *LMNA* promoter activity between the control group and the H_2_O_2_ group in 24 h and 48 h (Figure 4D). These results suggested that the augmentation of progerin, triggered by H_2_O_2_-induced oxidative damage, was potentially implicated in faulty alternative splicing. Additionally, the co-precipitation of LC3B with acetyl Lysine was increased in the H_2_O_2_-treated LSECs, and there was no difference in the co-precipitation of LC3B with progerin in the nucleus in the primary LSECs between the concurrent control group and the H_2_O_2_-treated group in 48 h (Figure 4E). The data suggested that a deficiency of progerin-related nucleophagic degradation might involve the excessive generation of progerin. Further, insufficient progerin-related nucleophagy probably was implicated in the high acetylation of nuclear LC3.

In addition, the RT-qPCR experiment showed that compared to the concurrent control group, the relative mRNA levels of Lamin B1 and Cav-1 were reduced in the H_2_O_2_-treated group in both 24 h and 48 h (Figure 4B). Moreover, the protein expression of Lamin B1 and Cav-1 in LSECs also was down-regulated due to H_2_O_2_ treatment (Figure 4C). In addition, the LC3 II/I protein expression in the nucleus and cytoplasm, the co-precipitation of LC3B with Lamin B1 and Cav-1 in the nucleus, and the co-precipitation of LC3B with Cav-1 in the cytoplasm in primary LSECs showed that H_2_O_2_ enhanced the interaction of LC3B with Lamin B1 in the nucleus rather than with Cav-1 in the nucleus in primary LSECs. Meanwhile, more Cav-1 co-precipitated with LC3B in the cytoplasm of H_2_O_2_-treated primary LSECs (Figure 4E,F), suggesting that Lamin B1 and Cav-1 were depleted by oxidative damage due to Lamin B1 nucleophagic degradation and Cav-1 autophagic degradation, aside from their reduced generation. Furthermore, the immunofluorescence displayed that more progerin co-localized with F-actin in the nucleus and perinuclear area of H_2_O_2_-treated primary LSECs, implying that the abnormal accumulation of progerin induced F-actin remodeling (Figure 4G).

Hence, these results suggested that NOX2- and NOX4-dependent oxidative damage provoked the abnormal accumulation of progerin, along with the loss of Lamin B1 and Cav-1, contributing to cytoskeleton remodeling and LSEC defenestration.

### 3.5. Antioxidants Promote Progerin Nucleophagic Degradation and Reverse Depletion of Lamin B1 and Cav-1 to Maintain LSEC Fenestrae

**Figure 5:**
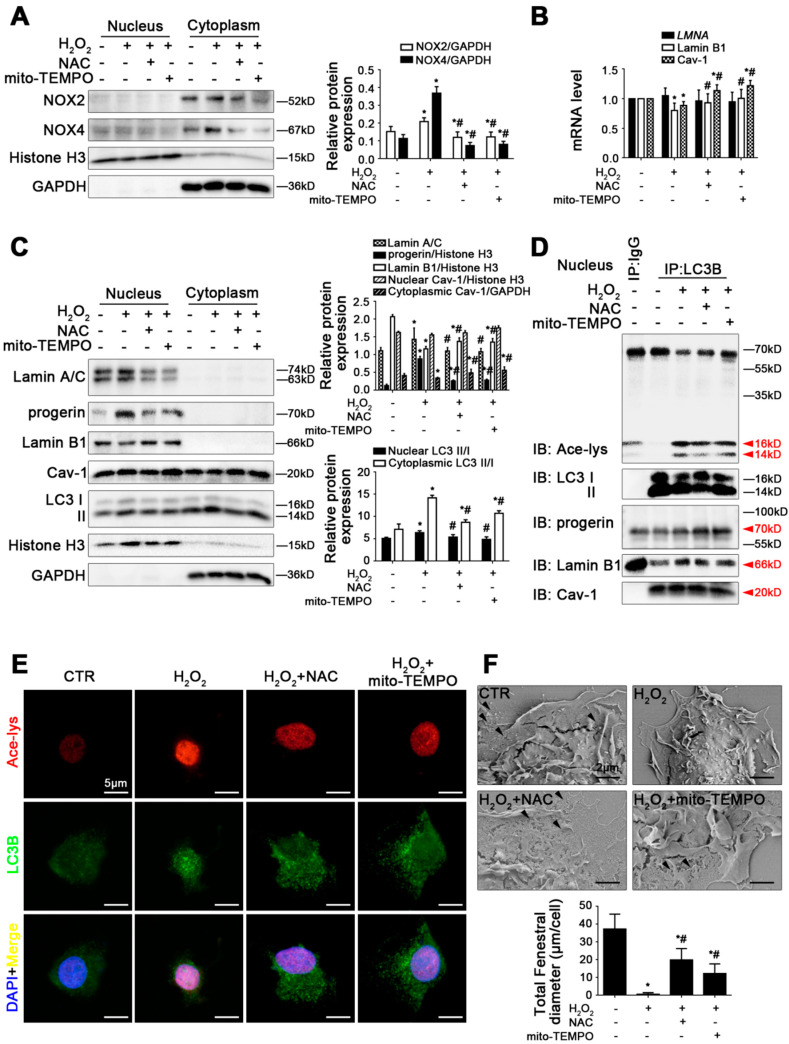
Antioxidants promote progerin nucleophagic degradation and reverse depletion of Lamin B1 and Cav-1 to maintain LSEC fenestrae. Fresh rat primary LSECs, isolated from normal male SD rats, were pre-treated with NAC (1 mM) or mito-TEMPO (100 U/mL) and stimulated with H_2_O_2_ (10 μM) in vitro for 48 h. (**A**) Representative immunoblots of NOX2 and NOX4 in nucleus and cytoplasm of primary LSECs in the four groups (CTR, H_2_O_2_, H_2_O_2_+NAC, H_2_O_2_+mito-TEMPO). The relative protein expression was quantified in the graph (right). * *p* < 0.05 versus the control group; ^#^ *p* < 0.05 versus the H_2_O_2_ group. (**B**) RT-qPCR analysis for mRNA levels of *LMNA*, Lamin B1, and Cav-1 of primary LSECs in the four groups. * *p* < 0.05 versus the control group; ^#^ *p* < 0.05 versus the H_2_O_2_ group. (**C**) Representative immunoblots of Lamin A/C, progerin, Lamin B1, Cav-1, and LC3 II/I in nucleus and cytoplasm of primary LSECs in the four groups. The relative protein expression and LC3 II/I expression in nucleus and cytoplasm are quantified in the two graphs (right). * *p* < 0.05 versus the control group; ^#^ *p* < 0.05 versus the H_2_O_2_ group. (**D**) The interaction of nuclear LC3B with acetyl Lysine, progerin, Lamin B1, and Cav-1 of primary LSECs was detected by the co-IP assay. Nuclear LC3B in primary LSECs was individually immunoprecipitated, and subsequently, acetyl Lysine, progerin, Lamin B1, Cav-1, and LC3 II/I in nucleus of primary LSECs were subjected to immunoblotting analysis. (**E**) The immunocytochemical co-localization of acetyl Lysine (red) with LC3B (green) of primary LSECs in the four groups (scale bar: 5 μm). Nuclei are shown by DAPI (blue). (**F**) The magnification of SEM for fenestrae in primary LSECs in the four groups (scale bar: 2 μm). The black triangles indicate fenestrae in LSECs. The total fenestral diameter is quantified in the graph (below). * *p* < 0.05 versus the control group; ^#^ *p* < 0.05 versus the H_2_O_2_ group.

Rat primary LSECs, pre-treated with antioxidants (including NAC and mito-TEMPO), were simulated with H_2_O_2_ for 48 h in vitro. As we expected, compared to the control group, the mRNA levels and the protein levels of NOX2 and NOX4, as well as the levels of ROS and mito-ROS, were enhanced in H_2_O_2_-treated LSECs, whereas these effects were relieved by NAC and mito-TEMPO (Figure 5A and Figure S5A,B). The mRNA level and the renilla luciferase reporter gene assay of *LMNA*, as well as the protein levels of Lamin A/C and progerin, showed that NAC and mito-TEMPO reduced H_2_O_2_-induced high expression of Lamin A/C and progerin in the nucleus, though they did not influence *LMNA* mRNA and its promoter activity. Moreover, NAC and mito-TEMPO enhanced the co-precipitation of LC3B with progerin in the nucleus of H_2_O_2_-treated LSECs, whereas less acetyl Lysine co-precipitated with LC3B in the nucleus of LSECs in the H_2_O_2_+NAC group and the H_2_O_2_+mito-TEMPO group (Figure 5B–D and Figure S5C). Indeed, the immunofluorescence showed that more acetyl Lysine co-localized with LC3B in the nucleus of H_2_O_2_-treated LSECs, and less acetyl Lysine with LC3B in the nucleus and LC3B was uniformly distributed throughout the cytoplasm. Meanwhile, more co-localization of progerin with LC3B was displayed in the nucleus and perinuclear area of LSECs in the NAC- or mito-TEMPO-treated group (Figure 5E and Figure S5E). These results indicated that antioxidants strengthen progerin nucleophagic degradation due to down-regulating acetylation of nuclear LC3.

In addition, the mRNA levels and the protein expression of Lamin B1 and Cav-1, as well as the co-precipitation of LC3B with Lamin B1 and Cav-1, showed that NAC and mito-TEMPO reversed the H_2_O_2_-induced loss of Lamin B1 and Cav-1 through enhancing the generation of Lamin B1 and Cav-1 and reducing their degradation (Figure 5B–D and Figure S5D). In reality, the magnification of SEM displayed that NAC and mito-TEMPO reversed H_2_O_2_-induced LSECs defenestration (Figure 5F). 

Consequently, antioxidants inhibited the NOX2- and NOX4-dependent oxidative damage to promote progerin nucleophagic degradation through reducing the acetylation of nuclear LC3 and recovering levels of Lamin B1 and Cav-1, contributing to maintaining LSEC fenestrae.

### 3.6. Excessive Autophagy Provokes Depletion of Lamin B1 and Cav-1, despite Activating Progerin Nucleophagic Degradation

**Figure 6:**
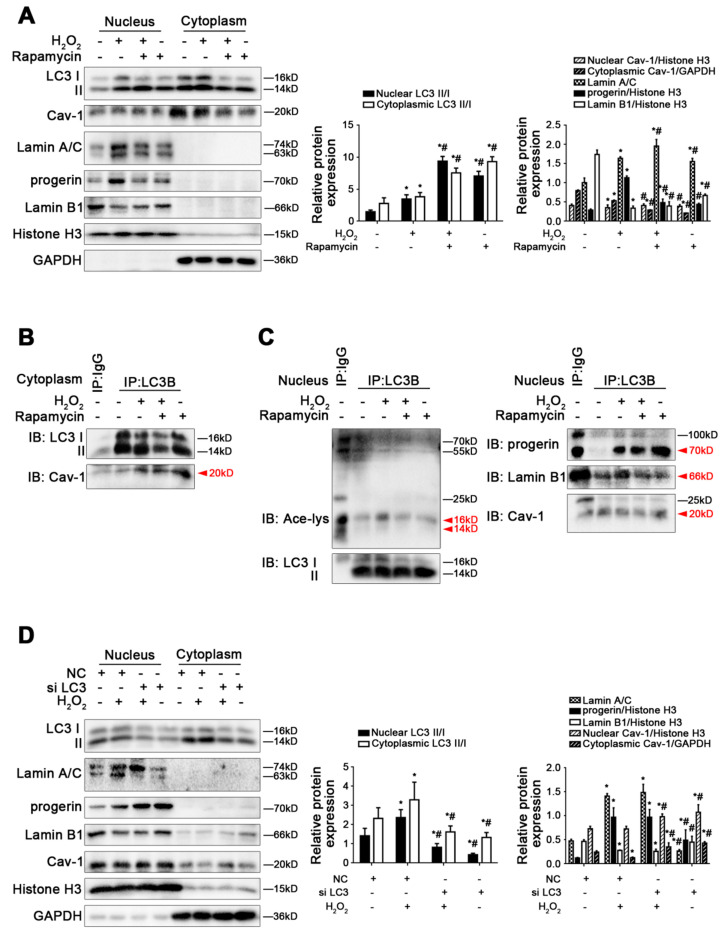
Excessive autophagy provokes depletion of Lamin B1 and Cav-1, as well as from progerin nucleophagic degradation. Fresh rat primary LSECs, isolated from normal male SD rats and cultured in vitro, were transfected with LC3B siRNA and nontarget siRNA (called NC) or pre-treated with rapamycin (10 nM) and then stimulated with H_2_O_2_ (10 μM) for 48 h. (**A**) Representative immunoblots of LC3 II/I, Cav-1, Lamin A/C, progerin, and Lamin B1 in nucleus and cytoplasm of primary LSECs in the four groups (CTR, H_2_O_2_, H_2_O_2_+rapamycin, rapamycin). The relative protein expression and LC3 II/I expression in nucleus and cytoplasm are quantified in the two graphs (right). * *p* < 0.05 versus the control group; ^#^ *p* < 0.05 versus the H_2_O_2_ group. (**B**) The interaction of cytoplasmic LC3B with Cav-1 was detected by the co-IP assay. Cytoplasmic LC3B in primary LSECs was individually immunoprecipitated, and subsequently, Cav-1 and LC3B in cytoplasm of primary LSECs in the four groups were subjected to immunoblotting analysis as indicated. (**C**) The interaction of nuclear LC3B with acetyl Lysine, progerin, Lamin B1, and Cav-1 was detected by the co-IP assay. Nuclear LC3B in primary LSECs was individually immunoprecipitated, and subsequently, acetyl Lysine, progerin, Lamin B1, Cav-1, and LC3 II/I in nucleus of primary LSECs in the four groups were subjected to immunoblotting analysis. (**D**) Representative immunoblots of LC3 II/I, Lamin A/C, progerin, Lamin B1, and Cav-1 in nucleus and cytoplasm of primary LSECs in the four groups (NC, NC+H_2_O_2_, si LC3+H_2_O_2_, si LC3). The relative protein expression and LC3 II/I expression in nucleus and cytoplasm are quantified in the two graphs (right). * *p* < 0.05 versus the NC group; ^#^ *p* < 0.05 versus the NC+H_2_O_2_ group.

To explore the effects of autophagy in cytoplasm and nucleophagy on oxidative stress-induced LSEC defenestration in depth, rat primary LSECs were pre-treated with rapamycin and then stimulated with H_2_O_2_ for 48 h. In our previous study, rapamycin might promote LSEC defenestration due to excessive autophagy [13,14]. The present research showed that rapamycin, an autophagy activator, reduced the protein levels of Lamin A/C, progeirn, Lamin B1 in the nucleus and cytoplasmic Cav-1 of H_2_O_2_-treated LSECs (Figure 6A). The co-precipitation of LC3B with Cav-1 in cytoplasm and the co-precipitation of LC3B with acetyl Lysine, progerin, Lamin B1, and Cav-1 in the nucleus of primary LSECs showed that rapamycin reinforced the interaction of LC3B with cytoplasmic Cav-1 instead of nuclear Cav-1, suggesting enhanced Cav-1 autophagic degradation in cytoplasm due to rapamycin. Moreover, rapamycin probably provoked progerin nucleophagic degradation due to down-regulating acetylation of nuclear LC3; meanwhile, rapamycin promoted Lamin B1 degradation (Figure 6B,C). The results indicated that rapamycin intensified the depletion of nuclear Lamin B1 and cytoplasmic Cav-1 to promote LSEC defenestration due to excessive autophagy, in spite of activating progerin-related nucleophagy. By contrast, neither the knockdown of LC3B with LC3B siRNA nor the inhibition of autophagy with 3-MA influenced the protein levels of progerin, Lamin A/C, and Lamin B1 in the nucleus, nor Cav-1 in the nucleus and cytoplasm of H_2_O_2_-treated LSECs (Figure 6D and Figure S6A). Additionally, the co-IP assay showed that 3-MA blocked the interaction of LC3B with Cav-1 in the cytoplasm, indicating a reduction in cytoplasmic Cav-1 degradation due to inhibiting autophagy. More acetyl Lysine co-precipitated with LC3B in the nucleus of H_2_O_2_-treated LSECs; meanwhile, the effect was strengthened by 3-MA. Further, there was no difference in the co-precipitation of LC3B with progerin, Lamin B1, or Cav-1 in the nucleus of primary LSECs between the H_2_O_2_ group and the H_2_O_2_+3-MA group (Supplementary Figure S6B,C).

The observations indicated that despite progerin nucleophagic degradation, excessive autophagy was unable to rescue oxidative damage-induced LSEC defenestration due to the loss of Lamin B1 and Cav-1.

### 3.7. Overexpression of SIRT1 Promotes Progerin Nucleophagic Degradation via Deacetylation of Nuclear LC3 and Inhibits Loss of Lamin B1 and Cav-1, Contributing to Maintaining LSEC Fenestrae

The above experimental evidence implied that progerin degradation and the protection of Lamin B1 and Cav-1 might be a new treatment for maintaining LSEC fenestrae, which was potentially implicated in regulating the acetylation of nuclear LC3. Rat primary LSECs were transfected with Flag-tagged SIRT1 adenovirus vector (called AV-SIRT1) or nontarget adenovirus vector (called AV-CTR) and then stimulated with H_2_O_2_ for 48 h. The mRNA levels and protein expression of SIRT1, NOX2, and NOX4, as well as the co-precipitation of LC3B with NOX2 and NOX4, showed that overexpressing SIRT1 with the adenovirus vector lessened H_2_O_2_-induced NOX2- and NOX4-dependent oxidative stress by reducing the production of NOX2 and NOX4 and enhancing their autophagic degradation (Supplementary Figure S7A–C).

**Figure 7:**
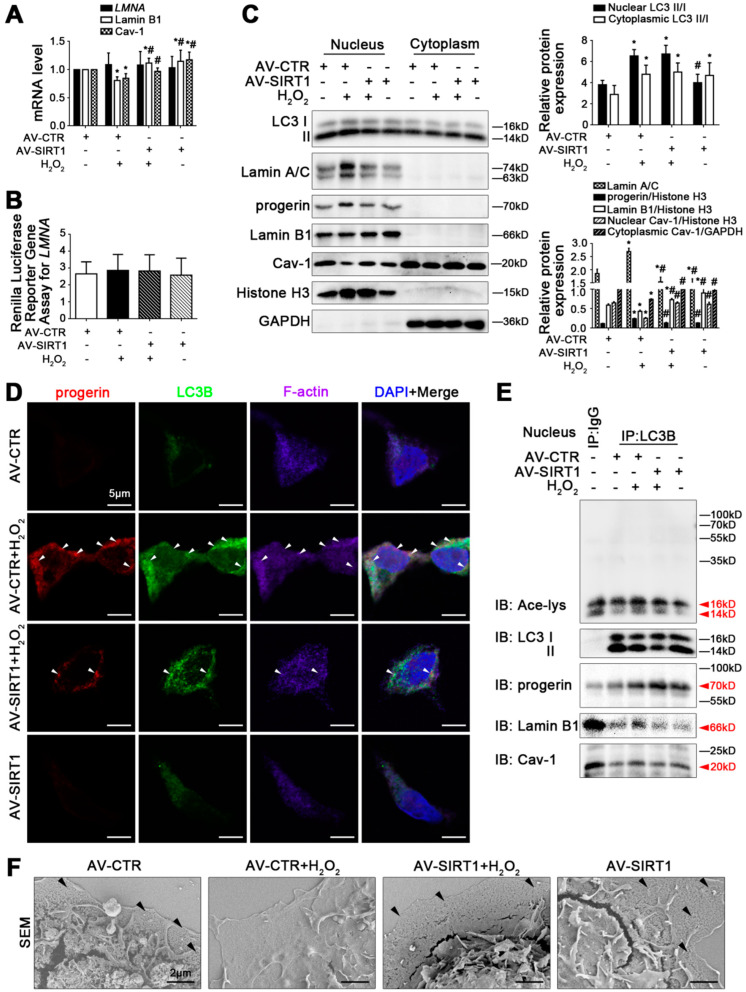
Overexpression of SIRT1 promotes progerin nucleophagic degradation via deacetylation of nuclear LC3 and inhibits loss of Lamin B1 and Cav-1, contributing to maintaining LSEC fenestrae. Fresh rat primary LSECs were transfected with SIRT1 adenovirus vector (called AV-SIRT1) to overexpress SIRT1 or nontarget adenovirus vector (called AV-CTR) and then stimulated with H_2_O_2_ (10 μM) for 48 h. (**A**) RT-qPCR analysis for mRNA levels of *LMNA*, Lamin B1, and Cav-1 of primary LSECs in the four groups (AV-CTR, AV-CTR+H_2_O_2_, AV-SIRT1+H_2_O_2_, AV-SIRT1). * *p* < 0.05 versus the AV-CTR group; ^#^ *p* < 0.05 versus the AV-CTR+H_2_O_2_ group. (**B**) Renilla Luciferase Reporter Gene Assay for *LMNA* promoter activity of primary LSECs in the four groups. (**C**) Representative immunoblots of LC3 II/I, Lamin A/C, progerin, Lamin B1, and Cav-1 in nucleus and cytoplasm of primary LSECs in the four groups. The relative protein expression and LC3 II/I expression in nucleus and cytoplasm are quantified in the two graphs (right). * *p* < 0.05 versus the AV-CTR group; ^#^ *p* < 0.05 versus the AV-CTR+H_2_O_2_ group. (**D**) The immunocytochemical co-localization of progerin (red) with LC3B (green) and F-actin (purple) of primary LSECs in the four groups (scale bar: 5 μm). Nucleus is shown by DAPI (blue). (**E**) The interaction of nuclear LC3B with acetyl Lysine, progerin, Lamin B1, and Cav-1 was detected by the co-IP assay. Nuclear LC3B in primary LSECs was individually immunoprecipitated, and subsequently, acetyl Lysine, progerin, Lamin B1, Cav-1 and LC3 II/I in nucleus of LSECs in the four groups were subjected to immunoblotting analysis. (**F**) The magnification of SEM for fenestrae in primary LSECs in the four groups (scale bar: 2 μm). The black triangles indicate fenestrae in LSECs.

The mRNA levels and the renilla luciferase reporter gene assay of *LMNA*, as well as the protein levels of Lamin A/C and progerin, showed that H_2_O_2_-induced high protein expression of Lamin A/C and progerin in the nucleus were down-regulated by overexpressing SIRT1 with the adenovirus vector, but *LMNA* mRNA and its promoter activity were unchanged (Figure 7A–C). The LC3B protein expression, the immunofluorescence, and the co-IP assay showed that the interaction of progerin with LC3B and F-actin, as well as the co-precipitation of LC3B with progerin, were enhanced by the overexpression of SIRT1 with the adenovirus vector, along with less co-precipitation of LC3B with acetyl Lysine and less co-localization of LC3B with acetyl Lysine (Figure 7C–E and Figure S7D). The data indicated that the overexpression of SIRT1 provoked progerin nucleophagic degradation and reversed F-actin remodeling via the deacetylation of nuclear LC3.

In addition, compared to the AV-CTR group, the mRNA levels of Lamin B1 and Cav-1 were reduced by H_2_O_2_, whereas this effect was reversed by the SIRT1 adenovirus vector (Figure 7A). In addition, as indicated in Figure 7C, compared to the AV-CTR group, the protein levels of Lamin B1 in the nucleus and Cav-1 in the nucleus and cytoplasm of H_2_O_2_-treated LSECs decreased, whereas this effect was recovered by overexpressing SIRT1 with the adenovirus vector. Meanwhile, the co-precipitation of LC3B with Lamin B1 and Cav-1 displayed that the overexpression of SIRT1 could inhibit Lamin B1-related nucleophagy and Cav-1-related autophagy (Figure 7E). The results suggested that overexpressing SIRT1 could promote the generation of Lamin B1 and Cav-1 and inhibit their degradation, contributing to reversing the loss of Lamin B1 and Cav-1. Indeed, the magnification of SEM showed that the overexpression of SIRT1 with the adenovirus vector maintained LSEC fenestrae (Figure 7F).

**Figure 8:**
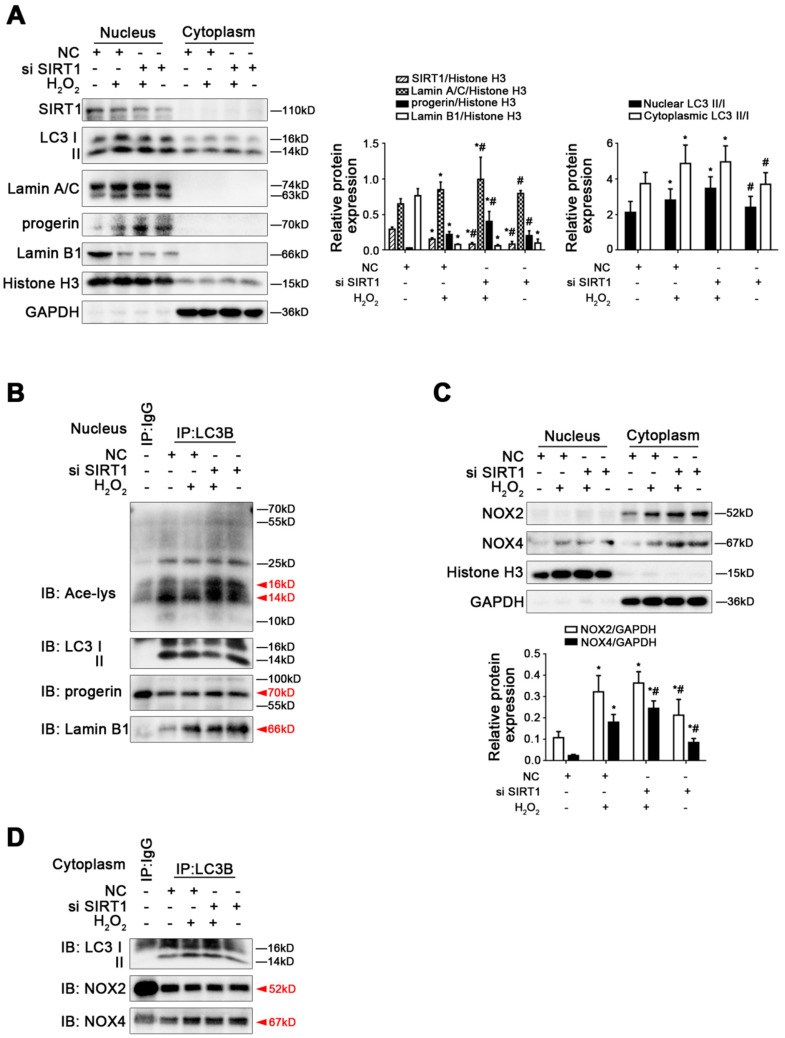
Knockdown of SIRT1 with siRNA activated Lamin B1 nucleophagic degradation but did not change H_2_O_2_-induced elevated progerin. Rat primary LSECs were transfected with SIRT1 siRNA or nontarget siRNA (called NC) and then treated with H_2_O_2_ (10 μM) for 48 h. The transfection efficiency was about 75%. (**A**) Representative immunoblots of SIRT1, LC3 II/I, Lamin A/C, progerin, and Lamin B1 in nucleus and cytoplasm of primary LSECs in the four groups (NC, NC+H_2_O_2_, si SIRT1+H_2_O_2_, si SIRT1). The relative protein expression and LC3 II/I expression in nucleus and cytoplasm are quantified in the two graphs (right). * *p* < 0.05 versus the NC group; ^#^ *p* < 0.05 versus the NC+H_2_O_2_ group. (**B**) The interaction of nuclear LC3B with acetyl Lysine, progerin, and Lamin B1 was detected by the co-IP assay. Nuclear LC3B in primary LSECs was individually immunoprecipitated, and subsequently, acetyl Lysine, progerin, Lamin B1, and LC3 II/I in nucleus of LSECs in the four groups were subjected to immunoblotting analysis. (**C**) Representative immunoblots of NOX2 and NOX4 in nucleus and cytoplasm of primary LSECs in the four groups. The relative protein expression is quantified in the graph (below). * *p* < 0.05 versus the NC group; ^#^ *p* < 0.05 versus the NC+H_2_O_2_ group. (**D**) The interaction of cytoplasmic LC3B with NOX2 and NOX4 was detected by the co-IP assay. Cytoplasmic LC3B in primary LSECs was individually immunoprecipitated, and subsequently, NOX2, NOX4, and LC3 II/I in cytoplasm of LSECs in the four groups were subjected to immunoblotting analysis.

By contrast, as indicated in Figure 8A,B, the protein expression of SIRT1, LC3 II/I, Lamin A/C, progerin, and Lamin B1, as well as the co-precipitation of LC3B with acetyl Lysine, progerin, and Lamin B1, demonstrated that the knockdown of SIRT1 with siRNA activated Lamin B1 nucleophagic degradation to lessen Lamin B1 expression due to enhancing Lamin B1-LC3 interaction. However, SIRT1 siRNA did not change the co-precipitation of LC3B with progerin in the nucleus of LSECs. In addition, the protein levels of NOX2 and NOX4, as well as the co-precipitation of LC3B with NOX2 and NOX4 in the cytoplasm of LSECs, revealed that silencing SIRT1 with siRNA did not influence the protein expression of NOX2 and NOX4 or the co-precipitation of LC3B with NOX2 and NOX4 (Figure 8C,D).

Hence, overexpressing SIRT1 initiated progerin nucleophagic degradation via the deacetylation of nuclear LC3 and subsequently relieved F-action remodeling and the depletion of Lamin B1 and Cav-1, contributing to reversing LSEC defenestration.

## 4. Discussion

**Figure 9:**
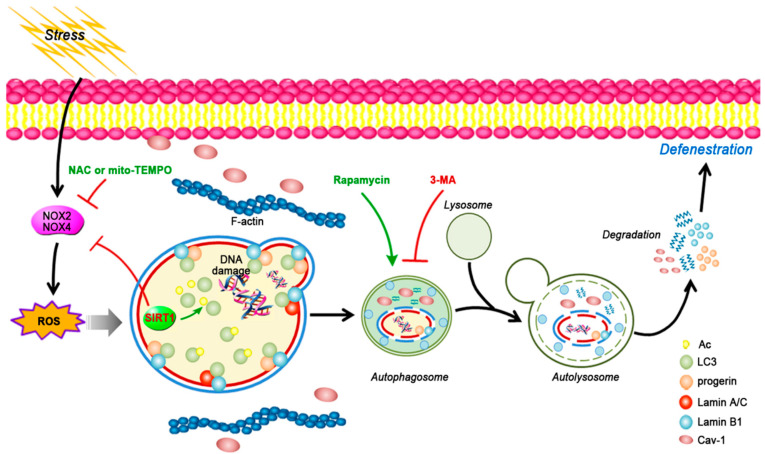
A schematic view of major signal pathways involved in the conclusion. Oxidative damage accelerates LSEC defenestration due to excessive accumulation of progerin and depletion of Cav-1. Overexpressing SIRT1 provokes progerin nucleophagic degradation via deacetylation of nuclear LC3 and inhibits depletion of Lamin B1 and Cav-1, contributing to reversing LSEC defenestration.

As described above, we explored the roles of autophagy in the cytoplasm and nucleophagy in LSEC phenotype and function in depth. Our study is the first to identify that SIRT1 provokes progerin-related nucleophagy and reverses the loss of Cav-1 to attenuate oxidative stress-induced LSEC defenestration via the deacetylation of nuclear LC3 (Figure 9). The principal findings include the following: (1) In vivo, during liver fibrogenesis, progerin abnormally accumulates in the nucleus of LSECs, with the loss of SIRT1 and Cav-1, whereas overexpressing SIRT1 with the adenovirus vector can reduce progerin and rescue Cav-1 degradation to attenuate LSEC defenestration and the first stage of liver fibrosis. (2) H_2_O_2_-induced oxidative damage accelerates LSEC defenestration due to the excessive accumulation of progerin and the depletion of Cav-1, whereas these effects are recovered by antioxidants. (3) Rapamycin aggravates H_2_O_2_-induced LSEC defenestration due to excessive Cav-1 autophagic degradation, despite elevated progerin-related nucleophagy. (4) Overexpressing SIRT1 provokes progerin nucleophagic degradation via the deacetylation of nuclear LC3 and inhibits the depletion of Cav-1, contributing to reversing LSEC defenestration.

Autophagy, a highly selective self-clearing process, degrades protein aggregates and impairs organelles, adaptively responding to detrimental stress [15]. It is of note that autophagy plays diverse roles in liver endothelium and liver diseases. For instance, in acute liver injury, autophagy is disordered in impaired endothelium, whereas moderate autophagy protects endothelial phenotype and liver function from oxidative stress [16]. We have previously found that severe autophagy promotes LSEC defenestration and liver fibrogenesis [13,14]. However, the major experimental observations on liver diseases focus on the autophagic degradation of cytoplasmic materials, with little attention to the role of autophagy in degrading nuclear components (namely nucleophagy). Nucleophagy selectively degrades impaired nuclear components, including damaged DNA or nuclear lamins, to exert crucial effects on the maintenance of nuclear structure and cellular homeostasis [17]. So far, nucleophagy is a hotspot for the prevention and treatment of premature aging and carcinoma. Emerging experimental evidence suggests that the degradation of damaged DNA via nucleophagy decelerates tumorigenesis; nonetheless, excessive nucleophagy, induced by persistent DNA damage, provokes cytotoxicity in normal cells through the apoptotic pathway [18,19]. In our previous research, we found that elevated autophagy emerges in LSECs in human fibrotic liver [13]. Here, our present study demonstrated the occurrence of autophagy and its enhancement in defenestrated LSECs during CCl_4_-induced liver fibrogenesis, in response to oxidative stress in vivo. Intriguingly, SIRT1 gene transfer to CCl_4_-induced liver fibrosis rat models could attenuate LSEC defenestration and liver fibrosis, despite enhanced autophagy being induced by overexpressing SIRT1. To confirm the interplay between SIRT1 and autophagy in LSEC fenestrae in depth, in vitro, we found that H_2_O_2_-induced oxidative damage accelerated LSEC defenestration, along with an increase in nucleophagy and autophagy in the cytoplasm. Meanwhile, activating autophagy, used by rapamycin, also promoted LSEC defenestration. However, overexpressing SIRT1 could maintain LSEC fenestrae, despite the augmentation of nucleophagy and autophagy in the cytoplasm. The results indicated that LSEC defenestration was not entirely determined by activating autophagy. The substrates of autophagic degradation potentially affected LSEC phenotype and function.

Different roles of nucleophagy depend on the substrate to be degraded. Nuclear lamins, composed of A-and B-type lamins, are essential for DNA repair, chromatin organization, and nuclear architecture [10,12]. Recent evidence confirms the degradation of nuclear lamina mediated by nucleophagy [17]. Furthermore, novel evidence shows that provoking the perturbation of nucleoskeleton and cell abnormal differentiation is implicated in nucleophagy, cellular premature senescence, and liver diseases [20,21]. The experimental findings show that in oncogenesis, lamins are nucleophagically degraded via directly interaction with LC3 in the nucleus [22]. More notably, the question should be asked as to which is the selective substrate of nucleophagy that is crucial for the maintenance of LSEC phenotype in liver fibrosis.

Progerin, a farnesylated mutant prelamin A, abnormally accumulates in cell nuclei due to premature senescence. Our previous study found that the aberrant generation of progerin, induced by premature senescence, could aggravate LSEC defenestration and liver fibrosis, whereas knockdown progerin could attenuate liver premature senescence [2,5]. In the present research, progerin was elevated in human liver fibrotic tissue and peripheral blood granulocytes of patients who were subjected to liver fibrosis. Moreover, in vivo, nuclear progerin abnormally accumulated in defenestrated LSECs during CCl_4_-induced liver fibrogenesis. In vitro, H_2_O_2_ induced the excessive generation of nuclear progerin to aggravate LSEC defenestration. Inhibiting progerin production or promoting its clearance may be a potential treatment for reversing LSEC defenestration.

It has been proposed that in response to DNA damage, Lamin A/C is a substrate of nucleophagy, which leaks nuclear DNA via an interaction with LC3 [23]. Another report reveals that progerin is involved in nucleophagy [24]. Surprisingly, our present study found that despite no change in the *LMNA* mRNA level and its promoter activity, H_2_O_2_-induced oxidative damage triggered the excessive accumulation of progerin, which was potentially implicated in faulty alternative splicing and/or a deficiency of progeirn clearance. Herein, our study mainly focused on the mechanism of progeirn clearance in response to oxidative damage. Antioxidants (including NAC and mito-TEMPO) and overexpressing SIRT1 with the adenovirus vector promoted progerin clearance through its nucleophagic degradation, rather than influencing *LMNA* mRNA and its promoter activity. Additionally, the emerging evidence reveals that the nucleophagic degradation of based Lamin B1 triggers nuclear breakdown and a standstill of canonical autophagy [17]. Hence, our observations indicated that insufficient progerin-related nucleophagy, provoked by oxidative stress, was probably attributed to the degradation of basal Lamin B1 and the acetylated form of nuclear LC3, contributing to the abnormal accumulation of progerin. An inference could be made on the deacetylation of nuclear LC3 being a promising novel approach for the maintenance of LSEC phenotype by promoting the clearance of progerin.

Additionally, Lamin B1, referred to as *LMNB1*, is abundantly expressed in the nucleus and assists in forming nuclear membrane, transcription factors, and chromatin, contributing to the stabilizion of the nucleoskeleton and cellular function. It has been proposed that the loss of Lamin B1 is triggered by severe oxidation and DNA damage, and the knockdown or degradation of Lamin B1 exacerbates nuclear architecture perturbations [25,26]. Similarly, we also showed that both the mRNA level and protein expression of Lamin B1 declined in defenestrated LSECs due to oxidative damage, along with strong Lamin B1-LC3 interaction, whereas antioxidants and the SIRT1 adenovirus vector could recover Lamin B1 level to maintain the nucleoskeleton and LSEC fenestrae by reducing oxidative damage and disrupting the Lamin B1-LC3 interaction. In addition, our previous and present studies indicate that rapamycin, a classical autophagy activator, aggravated oxidative stress-induced LSEC defenestration due to the excessive autophagic degradation of Lamin B1 and Cav-1, despite promoting progerin-related nucleophagy.

Cav-1, as a critical structural protein in LSEC fenestrae [27], is involved in maintaining LSEC phenotype and function. There are indications from us and others that the loss of Cav-1 could reduce the diameter of LSEC fenestration in response to stress or damage [14,28]. Indeed, we demonstrated that H_2_O_2_-induced Cav-1 autophagic degradation promoted LSECs defenestration, and these effects were exacerbated by rapamycin. However, antioxidants and the overexpression of SIRT1 could enhance Cav-1 production and inhibit Cav-1 autophagic degradation.

Generally, the autophagosomal membrane protein LC3 is mainly an acetylated form in the nucleus [29]. SIRT1, a histone deacetylase in sirtuin family proteins, is involved in nucleophagy and is beneficial for improving liver diseases. It has been evidenced that the deacetylation of LC3 by SIRT1 ensures the effective redistribution of LC3 from the nucleus to the cytoplasm, contributing to nucleophagy [29,30]. Our present study showed that SIRT1 was a multifunctional and pivotal hub in regulating nucleophagy and LSEC differentiation. The overexpression of SIRT1 with the adenovirus vector could inhibit NOX2- and NOX4-dependent oxidative stress and block Lamin B1-related nucleophagy and Cav-1-related autophagy, contributing to the recovery of the expression of Lamin B1 and Cav-1. On the other hand, the SIRT1-mediated deacetylation of nuclear LC3 could promote progerin nucleophagic degradation via strengthening the progerin-LC3 interaction. These effects alleviated LSEC defenestration and liver fibrosis.

Taken together, our research provides new insights into the effects of progerin-related nucleophagy in LSEC defenestration and suggests a new strategy for reversing LSEC defenestration and liver fibrosis by promoting progerin clearance via the SIRT1-mediated deacetylation of nuclear LC3.

However, there are still some limitations. The up-regulation of progerin expression and its stabilization were regulated by nuclear LC3 acetylation. In a future study, we will identify the sites of nuclear LC3 acetylation as the responder for stress-induced LSEC defenestration. Meanwhile, faulty alternative splicing of *LMNA* also might be implicated in the abnormal accumulation of progerin in the nucleus of defenestrated LSECs, which remains unexplored. Our next exploration will focus in depth on the nuclear LC3 acetylation and its regulation of the pre-mRNA splicing of *LMNA*.

## 5. Conclusions

In summary, SIRT1 drives the nucleophagic degradation of progerin mediated by the deacetylation of nuclear LC3 and improves the depletion of Lamin B1 and Cav-1, leading to the maintenance of LSEC fenestrae and the relief of liver fibrosis.

## Data Availability

Not applicable. The conclusions of the manuscript are based on relevant datasets available in the manuscript.

## Supplementary Materials

The following supporting information can be downloaded at: https://www.mdpi.com/article/10.3390/cells11233918/s1, Figure S1: Oxidative damage occurs in liver sinusoidal endothelial cells (LSECs) during CCl_4_-induced rat liver fibrogenesis; Figure S2: Overexpressing SIRT1 reduces progerin expression to improve CCl_4_-induced rat liver fibrosis; Figure S3: Progerin abnormally generates in defenestrated LSECs, along with depletion of Lamin B1 and Cav-1; Figure S4: H_2_O_2_ up-regulates NOX2- and NOX4-dependent oxidative damage to induce defenestration in LSECs; Figure S5: Antioxidants promote progerin-related nucleopahgy and prevent autophagic degradation of cytoplasmic Cav-1 via inhibiting oxidative damage; Figure S6: Inhibition of autophagy with 3-MA rescues Cav-1 level, rather than affects the levels of progerin and Lamin B1; Figure S7: Overexpressing SIRT1 inhibits oxidative stress-induced Cav-1 degradation.
