# Supplementary material for: Nucleophagic Degradation of Progerin Ameliorates Defenestration in Liver Sinusoidal Endothelium Due to SIRT1-Mediated Deacetylation of Nuclear LC3
Source: Cells. 2022 Dec 3;11(23):3918. doi: 10.3390/cells11233918 (PMC9738635; doi:10.3390/cells11233918)
Supplement: Supplementary file 1 [file cells-11-03918-s001.zip › cells-2027927-supplementary.pdf]

Supplementary

Supplementary methods

The following primer sequences were used for RT-qPCR. Human progerin primer (sense: 5′-GCGTCAGGAGCCCTGAGC-3′; antisense: 5′-GACGCAGGAAGCCTCCAC-3′). Rat NOX2 primer (sense: 5′-CATTTTCGTCAAGCGTCCCG-3′; antisense: 5′-AGTCGCCAACAATGCGGATA-3′). Rat NOX4 primer (sense: 5′-TTCGGGTGGCTTGTTGAAGT-3′; antisense: 5′-TGGGGTCCGGTTAAGACTGA-3′). Rat *LMNA* primer (sense: 5′-GCCTTCGCATCACTGAGTCT-3′; antisense: 5′-TCCTTGAACTCCTCACGCAC-3′). Rat Lamin B1 primer (sense: 5′-TTTCACAGAGCCGTCGTTCA-3′; antisense: 5′-GCGCACGTGAAGAGAAAGTC-3′). Rat Cav-1 primer (sense: 5′- GCGCCTTTCCCCCTCTATAC-3′; antisense: 5′-GGGCTTGTAGATGTTGCCCT-3′).

Supplementary figures and their legends

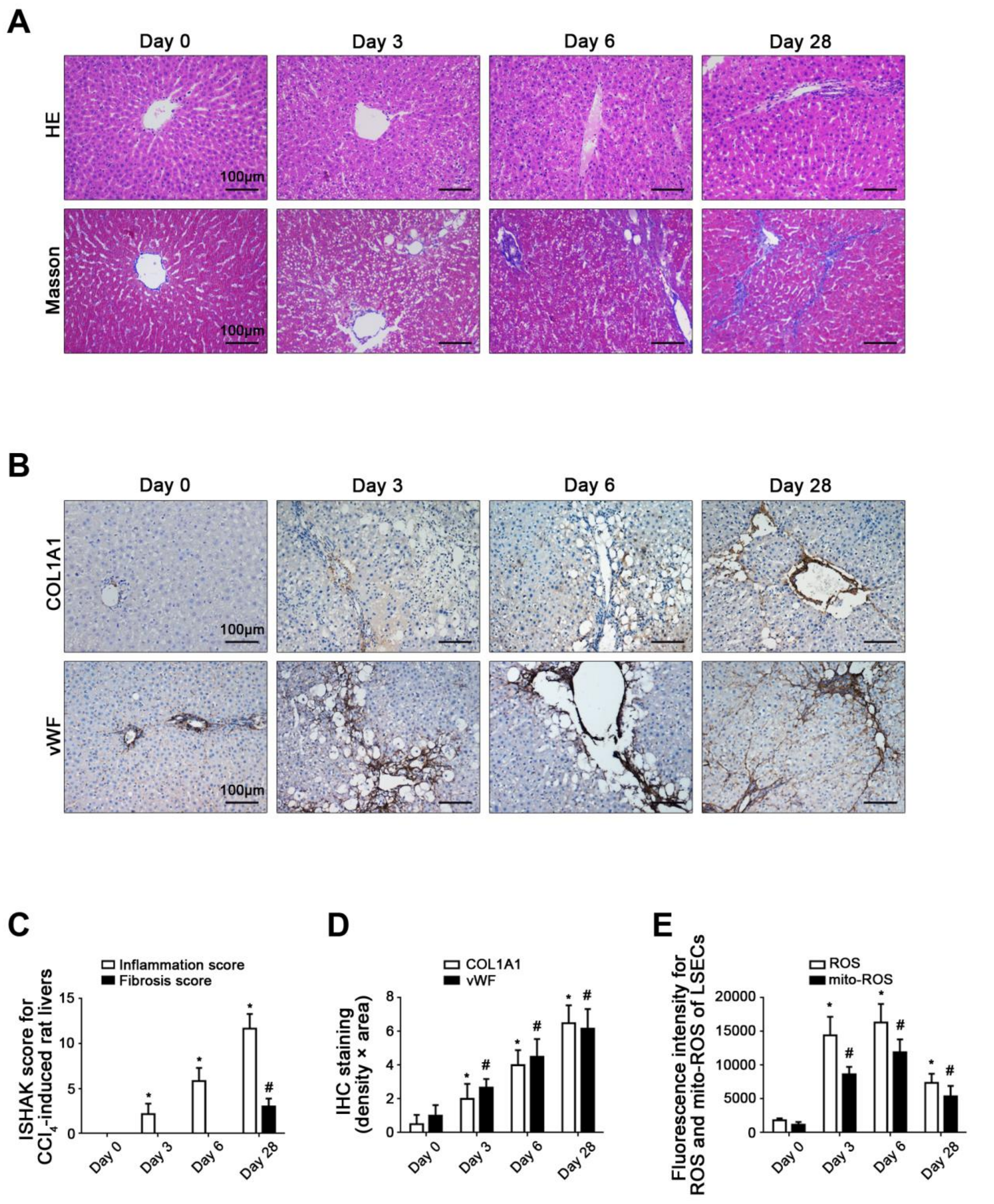

Supplementary Figure S1 Oxidative damage occurs in liver sinusoidal endothelial cells (LSECs) during CCl_4-induced rat liver fibrogenesis.

(A) Hematoxylin-eosin (HE) staining and Masson staining in liver tissue of CCl_4-induced rat models at different time points (Day 0, Day 3, Day 6, and Day 28) (Scale bar: 100 µm). **(B)** The immunohistochemical (IHC) staining of COL1A1 and vWF in liver tissue of CCl_4-induced rat models at different time points (Day 0, Day 3, Day 6, and Day 28) (Scale bar: 100 µm). **(C)** The quantified analysis of liver inflammation and fibrosis with ISHAK

score in the graph. *P<0.05 versus the inflammation score of the Day 0 group; #P<0.05 versus the fibrosis score of the Day 0 group. **(D)** The semi-quantified analysis for the area density of IHC staining of COL1A1 and vWF in the graph. *P<0.05 versus COL1A1 expression of the Day 0 group; #P<0.05 versus vWF expression of the Day 0 group. **(E)** Fluorescence intensity for reactive oxygen species (ROS) and mito-ROS of rat primary LSECs, which were isolated from CCl_4-induced rat models at different time points (Day 0, Day 3, Day 6, and Day 28). *P<0.05 versus ROS of the Day 0 group; #P<0.05 versus mito-ROS of the Day 0 group. N=6 per group.

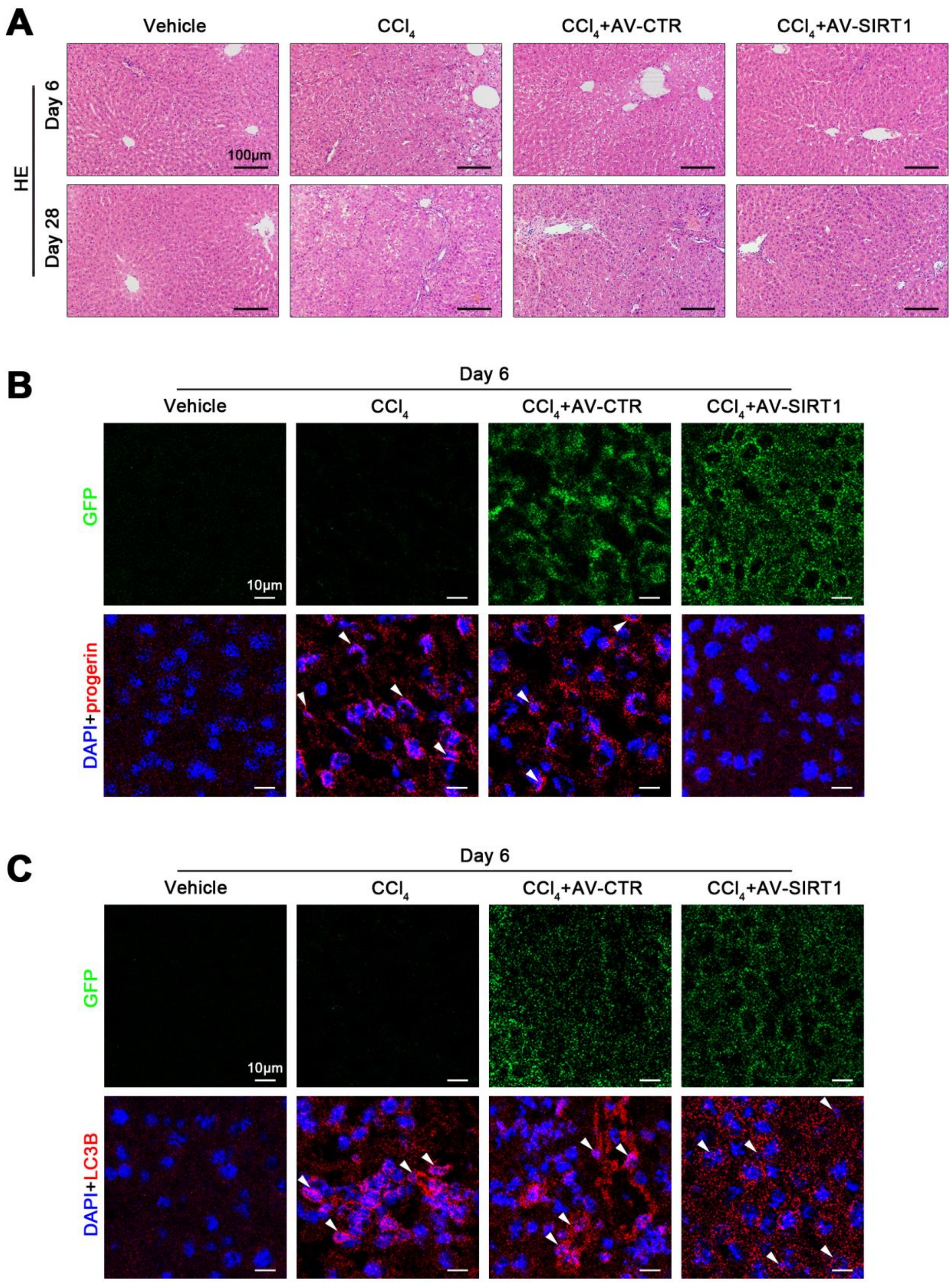

Supplementary Figure S2 Overexpressing SIRT1 reduces progerin expression to improve CCl_4-induced rat liver fibrosis.

(A) HE staining in liver tissue of CCl_4-induced rat models in the four groups (vehicle, CCl_4, CCl_4+AV-CTR, CCl_4+AV-SIRT1) on Day 6 and Day 28 (Scale bar: 100 μm). **(B)** The immunofluorescent (IF) staining for progerin (red) in liver tissue of CCl_4-induced rat models in the four groups on Day 6 (Scale bar: 10 μm). Adenovirus vectors were showed

by GFP (green). Nuclei were showed by DAPI (blue). **(C)** The IF staining for LC3B (red) in liver tissue of CCl_4-induced rat models in the four groups on Day 6 (Scale bar: 10 μm). Adenovirus vectors were showed by GFP (green). Nuclei were showed by DAPI (blue). N=12 per group.

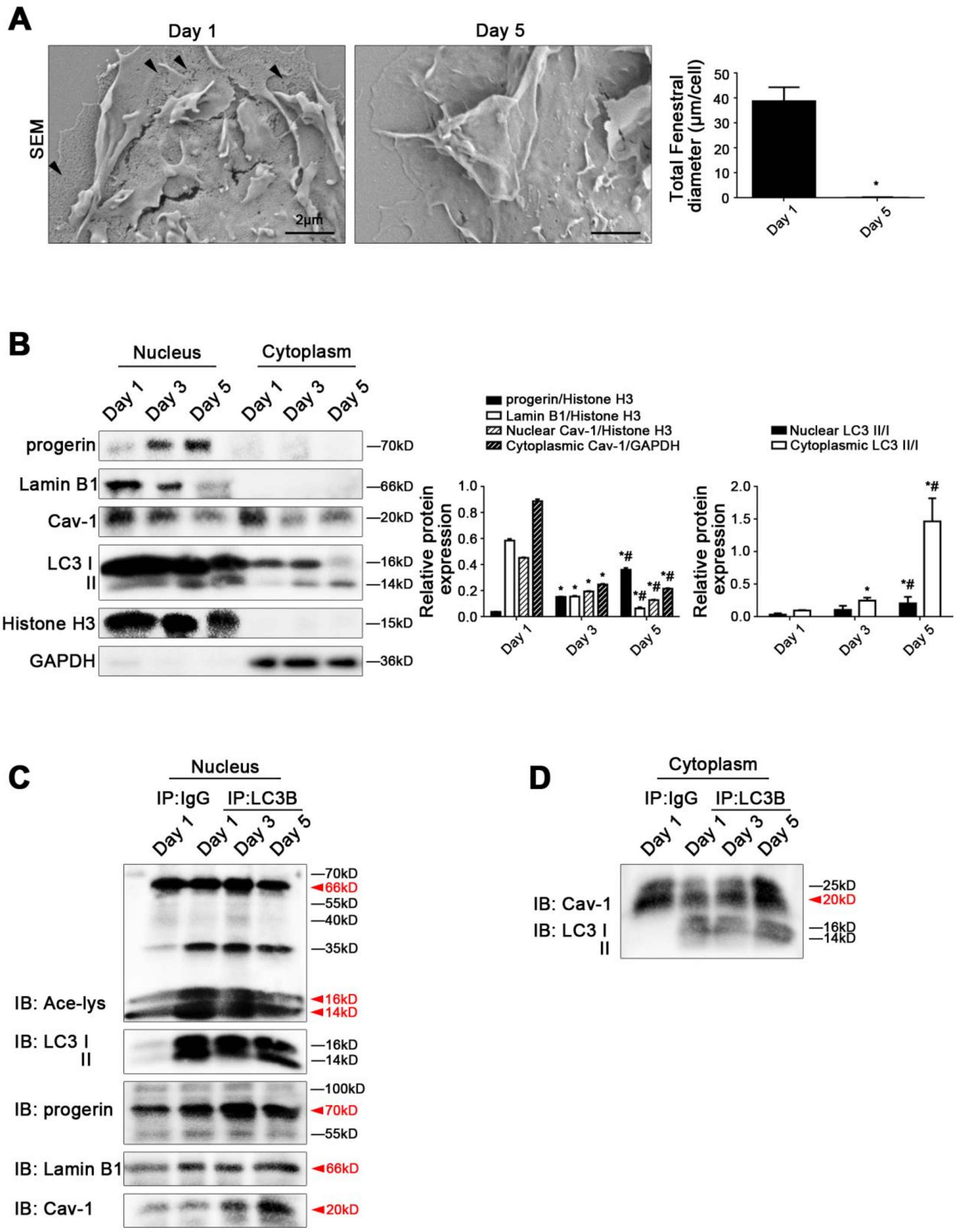

Supplementary Figure S3 Progerin abnormally generates in defenestrated LSECs, along with depletion of Lamin B1 and Cav-1.

Freshly rat primary LSECs were isolated from normal male SD rats and cultured *in vitro* for five days. **(A)** The magnification of SEM for fenestrae in primary LSECs on Day 1 and Day 5 (Scale bar: 2 μm). The black triangles indicated fenestrae in LSECs. The total fenestral diameter was quantified in the graph, right. **P*<0.05 versus the Day 1 group. **(B)** Representative immunoblots of progerin, Lamin B1, Cav-1, and LC3 II/I in nucleus and cytoplasm of primary LSECs on Day 1, Day 3, and Day 5. The relative protein expression and LC3 II/I expression in nucleus and cytoplasm were quantified in the two graphs (right). **P*<0.05 versus the Day 1 group; #*P*<0.05 versus the Day 3 group. **(C)** The interaction of nuclear LC3B with acetyl Lysine, progerin, Lamin B1, and Cav-1 was detected by the co-IP assay. Nuclear LC3B in primary LSECs was individually immunoprecipitated, and subsequently acetyl Lysine, progerin, Lamin B1, Cav-1, and LC3 II/I in nucleus of LSECs were subjected to immunoblotting analysis. **(D)** The interaction of cytoplasmic LC3B with Cav-1 was detected by the co-IP assay. Cytoplasmic LC3B in primary LSECs was individually immunoprecipitated, and subsequently Cav-1 and LC3 II/I in cytoplasm of LSECs were subjected to immunoblotting analysis.

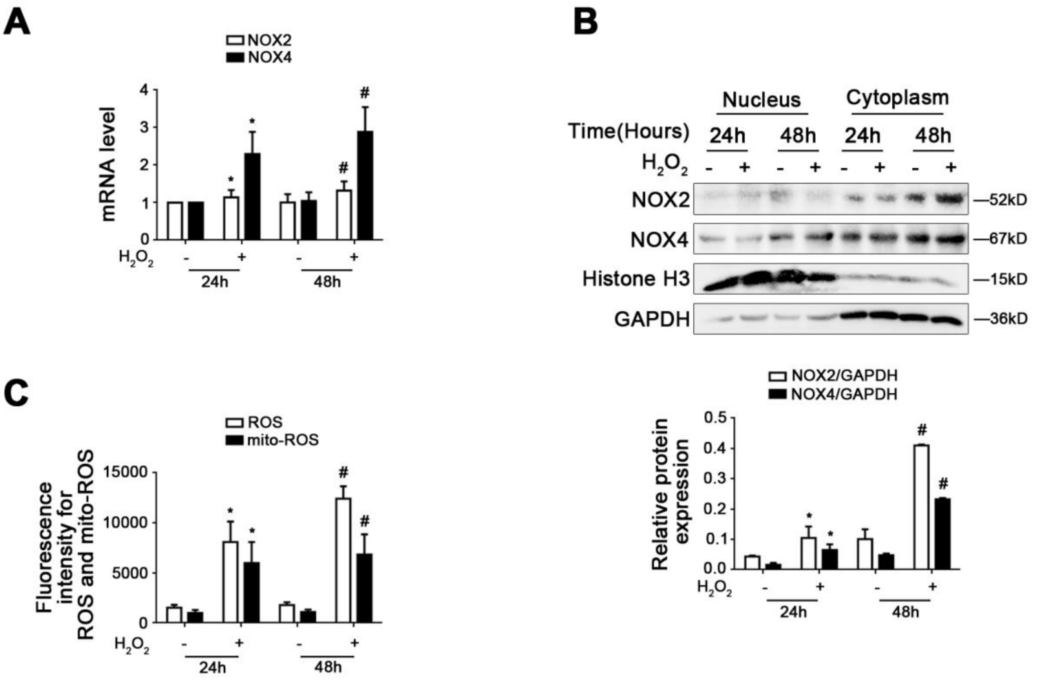

Supplementary Figure S4 H_2O_2 up-regulates NOX2- and NOX4-dependent oxidative damage to induce defenestration in LSECs.

Freshly rat primary LSECs, isolated from normal male SD rats, were treated with H_2O_2 (10 μM) *in vitro* for 48 hours. **(A)** RT-qPCR analysis for mRNA levels of NOX2 and NOX4 of primary LSECs in 24 hours and 48 hours. *$P<0.05$ versus the control group in 24 hours; $^{\#}P<0.05$ versus the control group in 48 hours. **(B)** Representative immunoblots of NOX2 and NOX4 in nucleus and cytoplasm of primary LSECs in 24 hours and 48 hours. The relative protein expression was quantified in the graph (below). *$P<0.05$ versus the control group in 24 hours; $^{\#}P<0.05$ versus the control group in 48 hours. **(C)** Fluorescence intensity for ROS and mito-ROS of primary LSECs in 24 hours and 48 hours. *$P<0.05$ versus the control group in 24 hours; $^{\#}P<0.05$ versus the control group in 48 hours.

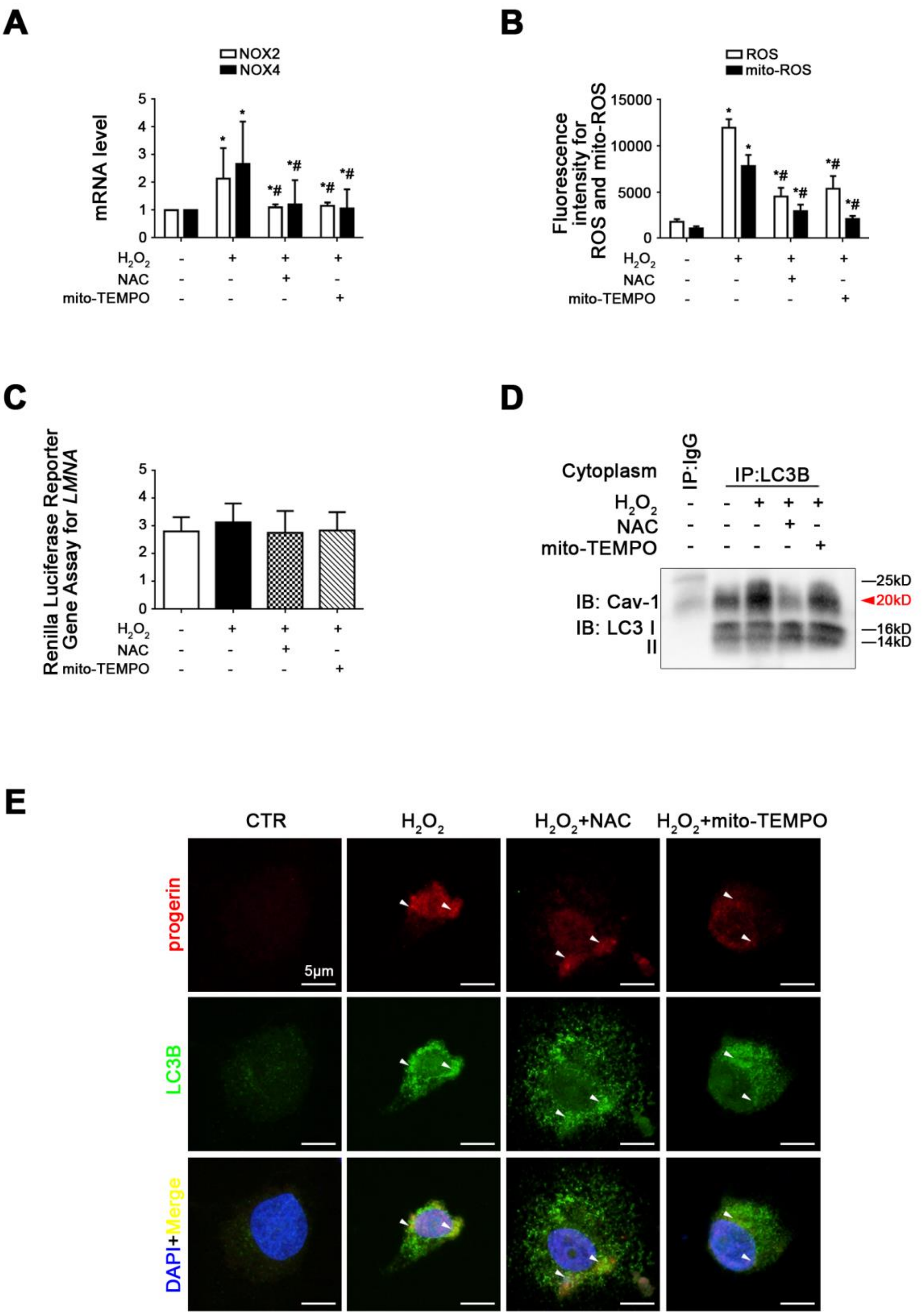

Supplementary Figure S5 Antioxidants promote progerin-related nucleopahgy and prevent autophagic degradation of cytoplasmic Cav-1 via inhibiting oxidative damage.

Freshly rat primary LSECs, isolated from normal male SD rats, were pre-treated with NAC (1 mM) or mito-TEMPO (100 U/ml), and stimulated with H_2O_2 (10 μM) *in vitro* for 48 hours. **(A)** RT-qPCR analysis for mRNA levels of NOX2 and NOX4 of primary LSECs in the four groups (CTR, H_2O_2, H_2O_2+NAC, H_2O_2+mito-TEMPO). **P*<0.05 versus the

control group; $^{\#}P<0.05$ versus the H_2O_2 group. **(B)** Fluorescence intensity for ROS and mito-ROS of primary LSECs in the four groups. $^{*}P<0.05$ versus the control group; $^{\#}P<0.05$ versus the H_2O_2 group. **(C)** Renilla Luciferase Reporter Gene Assay for *LMNA* promoter activity of primary LSECs in the four groups. $^{*}P<0.05$ versus the control group; $^{\#}P<0.05$ versus the H_2O_2 group. **(D)** The interaction of cytoplasmic LC3B with Cav-1 was detected by the co-IP assay. Cytoplasmic LC3B in primary LSECs was individually immunoprecipitated, and subsequently Cav-1 and LC3 II/I in cytoplasm of LSECs were subjected to immunoblotting analysis. **(E)** The immunocytochemical co-localization of progerin (red) with LC3B (green) of primary LSECs in the four groups (Scale bar: 5 μm). Nuclei were showed by DAPI (blue).

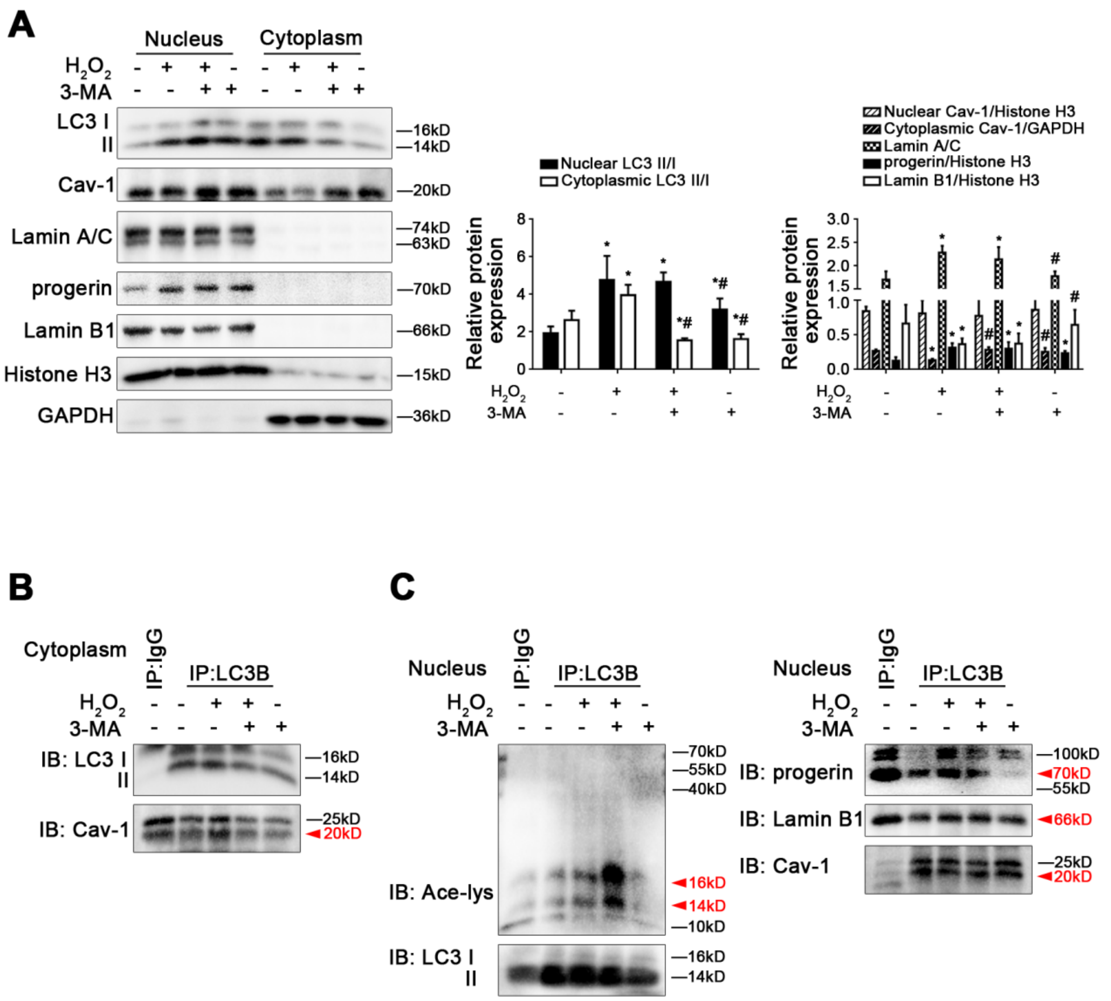

Supplementary Figure S6 Inhibition of autophagy with 3-MA rescues Cav-1 level, rather than affects the levels of progerin and Lamin B1.

Freshly rat primary LSECs, isolated from normal male SD rats, were pre-treated with rapamycin (10 nM) and stimulated with H_2O_2 (10 μM) for 48 hours. **(A)** Representative immunoblots of LC3 II/I, Cav-1, Lamin A/C, progerin, and Lamin B1 in nucleus and cytoplasm of primary LSECs in the four groups (CTR, H_2O_2, H_2O_2+3-MA, 3-MA). The relative protein expression and LC3 II/I expression in nucleus and cytoplasm were quantified in the two graphs (right). $^{*}P<0.05$ versus the control group; $^{\#}P<0.05$ versus the H_2O_2 group. **(B)** The interaction of cytoplasmic LC3B with Cav-1 was detected by the co-IP assay. Cytoplasmic LC3B in primary LSECs was individually immunoprecipitated, and subsequently Cav-1 and LC3 II/I in cytoplasm of LSECs in the four groups were subjected to immunoblotting analysis. **(C)** The interaction of nuclear LC3B with acetyl Lysine, progerin, Lamin B1, and Cav-1 was detected by the co-IP assay. Nuclear LC3B in primary LSECs was individually immunoprecipitated, and subsequently acetyl Lysine, progerin, Lamin B1, Cav-1, and LC3 II/I in nucleus of primary LSECs in the four groups were subjected to immunoblotting analysis.

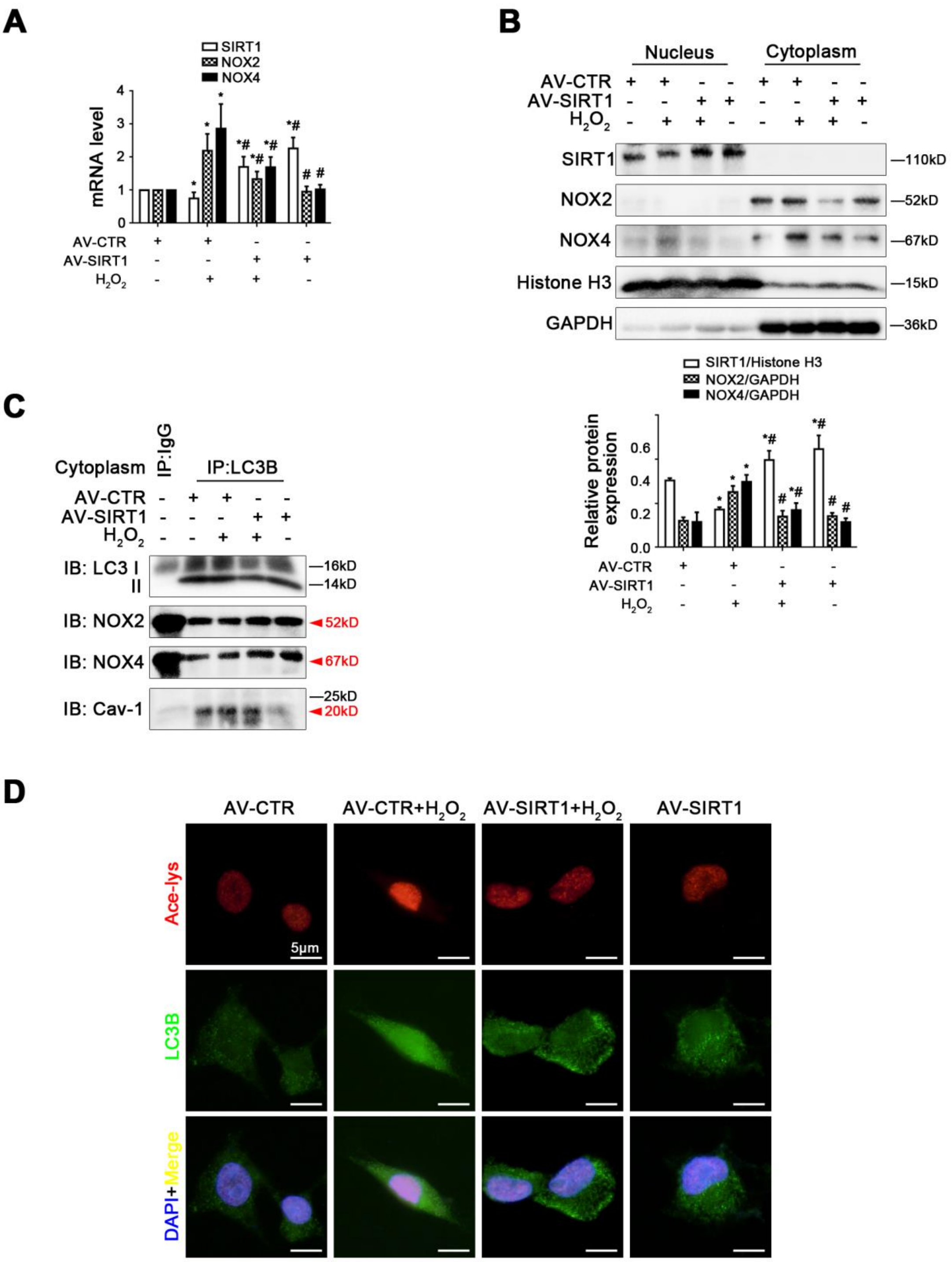

Supplementary Figure S7 Overexpressing SIRT1 inhibits oxidative stress-induced Cav-1 degradation.

Freshly rat primary LSECs were transfected with SIRT1 adenovirus vector (called AV-SIRT1) to overexpress SIRT1 or nontarget adenovirus vector (called AV-CTR), and then stimulated with H_2O_2 (10 μM) for 48 hours. **(A)** RT-qPCR analysis for mRNA levels of SIRT1, NOX2, and NOX4 of primary LSECs in the four groups (AV-CTR,

AV-CTR+H_2O_2, AV-SIRT1+H_2O_2, AV-SIRT1). *P<0.05 versus the AV-CTR group; #P<0.05 versus the AV-CTR+H_2O_2 group. **(B)** Representative immunoblots of SIRT1, NOX2, and NOX4 in nucleus and cytoplasm of primary LSECs in the four groups. The relative protein expression was quantified in the graph (below). *P<0.05 versus the AV-CTR group; #P<0.05 versus the AV-CTR+H_2O_2 group. **(C)** The interaction of cytoplasmic LC3B with NOX2, NOX4, and Cav-1 was detected by the co-IP assay. Cytoplasmic LC3B in primary LSECs was individually immunoprecipitated, and subsequently NOX2, NOX4, Cav-1, and LC3 II/I in cytoplasm of primary LSECs in the four groups were subjected to immunoblotting analysis. **(D)** The immunocytochemical co-localization of acetyl Lysine (red) with LC3B (green) of primary LSECs in the four groups (Scale bar: 5 μm). Nuclei were showed by DAPI (blue).
